# Defect-Engineered Graphene Nanoribbons for Enhanced DNA Sequencing: A Study of Structural Defects and Their Impact on Nucleobase Interaction and Quantum Transport

**DOI:** 10.1021/acs.jpcb.5c03247

**Published:** 2025-09-22

**Authors:** Rameshwar L. Kumawat, Sanjiv K. Jha, Benjamin O. Tayo, C. David Sherrill

**Affiliations:** † School of Chemistry and Biochemistry, 3270 Georgia Institute of Technology, Atlanta, Georgia 30332, United States; ‡ Mathematics and Science Division, 31705 GateWay Community College, Maricopa Community Colleges, Phoenix, Arizona 85034, United States; § School of Engineering, 565519 University of Central Oklahoma, Edmond, Oklahoma 73034, United States

## Abstract

Graphene, a low-dimensional material, has shown significant promise in bioelectronics over the past two decades. Most research in this field has focused on pristine graphene. However, experimentally fabricated two-dimensional (2D) graphene and one-dimensional (1D) graphene nanoribbons (GNRs) often contain impurities, such as Stone–Wales (sw) and divacancy (dv) defects. In this study, we conducted a comparative analysis of the adsorption behavior of DNA nucleobases–adenine (A), guanine (G), thymine (T), and cytosine (C)—on three types of graphene nanoribbon (GNR) surfaces: pristine (prGNR), divacancy-defected (dvGNR), and Stone–Wales-defected (swGNR). Using semilocal (PBE) and van der Waals-corrected density functional theory methods (vdW-DF2 and PBE-D2), we evaluated the binding energies of the nucleobases on the different GNR surfaces. Our results show that defected GNRs exhibit less negative binding energies for all nucleobases compared to prGNR when dispersion interactions are taken into account. The binding energies calculated using PBE, PBE-D2, and vdW-DF2 methods range from −0.06 to −0.10, −0.55 to −0.80 and −0.59 to −0.78 eV, respectively. The vdW-DF2 method effectively captures vdW interactions, with binding energies following the order G > A > T > C. These interactions result in weak binding between nucleobase and the π-states of the GNR surfaces, inducing a small interfacial dipole and a shift in the energy bandgap. Quantum transport analysis reveals that while pristine GNRs exhibit distinct conduction channels, defects—such as dv and sw configurations—introduce localized states that interact with delocalized ones, generating pronounced *Fano resonances* characterized by sharp dips in the transmission spectra. Physisorption of DNA nucleobases on different GNR surfaces induces unique resonance peaks in the transmission function, influenced by the type and position of defects. Conductance sensitivity analysis indicates prGNR as a promising candidate for nucleobase detection, leveraging *Fano resonances* for precise electronic measurements. However, defected GNRs also exhibit significant sensitivity. Furthermore, Current–Voltage (*I*–*V*) analysis identifies dvGNR devices as the most effective for nucleobase detection due to their high current sensitivity and distinct responses across nucleobases. While prGNR devices detect certain nucleobases, they show less consistent performance due to uniform current trends at higher biases. In contrast, swGNR devices effectively differentiate all four nucleobases through distinct current signals in the 0.6–0.8 V range. These findings underscore the potential of defect-engineered GNRs for the next-generation DNA sequencing applications.

## Graphical abstract

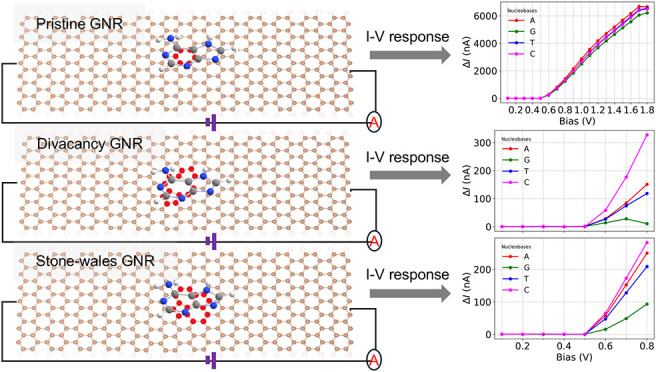

## 1 Introduction

With the advent of nanotechnology, low-dimensional materials such as graphene have been discovered and synthesized.^1,2^ The experimental realization of graphene has spurred significant research due to its potential revolutionary impacts across various industries. Graphene, a two-dimensional material, is composed of a single layer of sp^2^-bonded carbon (C) atoms arranged in a honeycomb lattice.^3^ It boasts exceptional chemical, electrical, mechanical, optical, and quantum transport properties.^3,4^ However, these diverse applications require graphene to have tunable and controllable properties, which can be achieved through modifications. One challenge is the absence of a bandgap in graphene, which must be addressed to attain a high *I*_on_/*I*_off_ current ratio in graphene-based field-effect transistor devices.^5^

In this context, controlled defect engineering in sp^2^ C-based materials has emerged as a topic of significant interest and excitement.^6^ Indeed, the electronic and transport properties of graphene-based materials can be significantly enhanced through chemical modifications, including substitution, molecular doping, and functionalization.^7–10^ Another approach to tuning graphene’s properties involves the introduction of structural defects (e.g., vacancies) via ion irradiation, electron irradiation, or scanning tunneling microscopy (STM), which can be achieved at the nanoscale in sp^2^ C-based structures.^11–13^

Various types of defects can occur in graphene, and they are known to significantly alter its properties. The presence of structural defects disrupts the material’s perfect symmetry, opening a new area of research focused on understanding the effects of these defects on graphene’s properties. Some studies have shown that defects can be beneficial, such as in chemical and electrochemical applications where they create preferential sites for the adsorption of atoms and molecules, which can be utilized in liquid and gas sensing.^11,14^ Among the different types of defects, Stone–Wales (sw) defects^15^ and 585 or divacancy (dv) defects are particularly noteworthy.^16^ The sw defect involves the rotation of a C–C bond by 90°, leading to the formation of two pentagons and two heptagons, which can influence the material’s mechanical and electronic properties.^16–18^ The 585 defect,^19^ consisting of alternating pentagons and octagons, also alters the electronic structure of graphene, affecting its transport properties. These defects are crucial in understanding the behavior of graphene in various applications, including biosensing. During experimental studies, defects can form unintentionally or be deliberately introduced. Their presence is critical to consider because they can significantly impact the material’s properties and its suitability for specific applications. In biosensing, for example, defects in graphene nanoribbons (GNRs) can influence the interaction between DNA molecules and the GNR surface.^20,21^ The presence of defects may enhance or diminish the sensitivity and accuracy of DNA detection, making it essential to understand and control defect formation for optimizing biosensor performance.

DNA sequencing, which provides crucial genomic information for diagnosing, treating, and preventing diseases, can benefit from nanoscale devices that thread single-strand (ss) DNA through a nanopore, nanogap, and nanochannel.^21–28^ However, challenges such as controlling DNA translocation speed, suppressing stochastic nucleobase motions, and resolving signal overlap between different nucleobases must be addressed to make this approach practical.^27,29,30^ Understanding the interactions between DNA nucleobases and inert surfaces of low-dimensional materials is therefore of significant interest for single-molecule detection. Various 2D and 1D materials, including graphene,^22,31–35^ silicene,^36^ boron nitride (BN),^37,38^ boron carbide,^24^ Ti_3_C_2_ MXene,^39^ phosphorene,^40–42^ and cabron nanotubes^43^ have been proposed for nucleobase recognition. Among these, graphene surfaces, both 2D and 1D, have been particularly well-studied.

For instance, Kim et al.^22^ theoretically demonstrated that an ultrafast and highly sensitive GNR nanochannel device could electrically distinguish all four nucleobases by controlling their motion through π–π interactions and deciphering their *Fano resonance*-driven transmission characteristics. Similarly, Thomas et al.^37^ showed that individual nucleobase detection could be achieved through transmission measurements using narrow semiconducting nanoribbons like graphene, MoS_2_, silicene, and hexagonal BN, which exhibit dips in the transmission curve when interacting with nucleobases. Amorim et al.^36^ also proposed a 2D silicene-based device as an electrical biosensor. Despite the potential of low-dimensional materials for these applications, challenges persist, as detailed in our review article,^21^ hindering the practical use of these materials in DNA sequencing. Although graphene’s atomically thin membrane and strong C–C bonds make it a promising candidate, researchers continue to seek more sensitive materials for DNA sequencing.

Herein, we chose to work with a special type of graphene nanoribbon, called a semiconducting armchair graphene nanoribbon (aGNR), because it has a tunable bandgap and excellent transport properties–both of which are crucial for achieving high-resolution signals in DNA sequencing. Another reason for this choice is that aGNRs can now be fabricated with great precision using bottom-up synthesis techniques, which allow control over their width and edge structure.^44^ Motivated by these advantages, we designed a study to compare GNR nanochannel devices with and without defects, to find a configuration best suited for DNA sequencing. We focused on GNR devices that can be built through bottom-up methods, examining how structural defects influence the interaction between the four DNA bases–adenine (A), guanine (G), cytosine (C), and thymine (T).^45,46^ To understand this interaction in detail, we used three computational approaches–PBE, PBE-D2, and vdW-DF2–that account for different aspects of the forces between atoms. We also performed quantum transport simulations by combining density functional theory (DFT) with the nonequilibrium Green’s function (NEGF) method, to evaluate how these GNR devices would perform. As a single-stranded DNA (ssDNA) strand moves through the GNR nanochannel, each base interacts with different types of GNRs–prGNR, dvGNR, and swGNR–through π–π stacking. These interactions cause changes in the electronic transmission of the device at specific energies due to *Fano resonance*, which helps in distinguishing between different nucleobases. Our analysis of the electronic transmission and current response shows that each type of GNR responds differently to each nucleobase, offering a way to accurately identify the DNA bases. This suggests that GNR nanochannel devices–especially when tailored with or without defects–can be highly effective for DNA sequencing.

## 2 Computational Methods

### 2.1 Electronic Structure Calculations

The structural relaxation of the graphene nanoribbon (GNR) surfaces and nucleobases was performed using plane-wave density functional theory (DFT) as implemented in the QUANTUM ESPRESSO package.^47,48^ We used the semilocal PBE^49^ functional, both with and without Grimme’s D2 dispersion correction,^50,51^ to evaluate the role of van der Waals interactions. For comparison, we also employed the nonlocal vdW-DF2 functional,^52^ which includes its own exchange and correlation components. Unlike dispersion-corrected PBE, vdW-DF2 does not rely on PBE as a base; instead, it incorporates nonlocal correlation terms designed to capture van der Waals interactions from first-principles, without the need for empirical corrections. Accounting for dispersion is particularly important in these systems due to the presence of π–π stacking interactions. All geometries were optimized using PBE, PBE-D2, and vdW-DF2 functionals. The optimized structures were then used to calculate the interaction energies. We selected the Grimme-D2 dispersion correction over the D3 and D4 variants because it remains widely used, particularly in studies of large systems such as graphene and metal interfaces,^53–55^ where computational efficiency is a key consideration. In addition, our primary objective is to compare the relative adsorption trends among different nucleobases rather than to determine highly accurate binding energies. For such comparative analyses, the D2 correction has been demonstrated to provide reliable and consistent results.

Initial geometry relaxation was conducted using a variable-cell optimization scheme, allowing both atomic positions and cell parameters to relax. Geometry optimization was performed using the Broyden-Fletcher-Goldfarb-Shanno (BFGS) algorithm^56–59^ for both atomic and cell dynamics. The energy cutoff for the plane-wave basis set was set to 45 Ry, and the charge density cutoff was set to 450 Ry. The convergence threshold for the self-consistent field (SCF) calculations was set to 1 × 10^–7^ Ry. Structures were fully relaxed until the components of the Hellmann–Feynman forces acting on each atom were less than 1 × 10^–3^ Ry/Bohr. Due to the large size of our systems, geometry optimization calculations were performed using a 1 × 1 × 2 *k*-point grid. Band structure calculations were carried out using a denser 1 × 1 × 6 *k*-point grid. Ultrasoft pseudopotentials (USPP)^60^ were employed for all calculations. We used 20 Å of vacuum in the x*y*-directions to avoid the interactions between periodic images of the supercell. All computations performed here are obtained in the vacuum (gas) phase, which is sufficient to quantify the intrinsic interactions between the nucleobases and GNRs. In condensed phases, additional effects arise from the surrounding environment (e.g., interactions with water molecules, nearby nucleobases, and the DNA backbone), but these are highly system-dependent. Importantly, such effects are not expected to fundamentally modify the direct nucleobase-GNR interactions.

### 2.2 Quantum Transport Calculations

The quantum transport properties were calculated within the Landauer-Büttiker formalism using the nonequilibrium Green’s function (NEGF) approach combined with DFT, as implemented in the TranSIESTA package.^18,61–65^ For this purpose, the transport system is divided into three main parts: left (lead + buffer region), device region, and right (buffer region + lead), as shown in Figure S1 (Supporting Information). The vdW-DF functional,^52,66–68^ combined with a DZP basis set and a 1 × 1 × 100 *k*-point grid, is used for all transport calculations. The transmission as a function of energy is computed using the following eq (eq 1)­

1 $$T\left(E,V_{b}=0\right)=\Gamma_{L}\left(E,V\right)G\left(E,V\right)\Gamma_{R}\left(E,V\right)G^{†}\left(E,V\right)$$

where Γ_L_(*E,V*) and Γ_R_(*E,V*) are the coupling matrices of the left (L) and right (R) leads, respectively, and *G*(*E*,*V*) and *G* ^†^(*E*,*V*) represent the retarded and advanced Green’s functions at zero bias.

The electric current *I*(*V*_b_) under an applied bias can be expressed in the Landauer-Büttiker form (eq 2)­

2 $$I\left(V_{b}\right)=\frac{2e}{h}\int_{\mu_{R}}^{\mu_{L}}T\left(E,V_{b}\right)\left[f\left(E-\mu_{L}\right)-f\left(E-\mu_{R}\right)\right]dE$$

where *f*(*E* – μ_L_) and *f*(*E* – μ_R_) are the Fermi–Dirac functions for electrons in the L and R leads with the respective chemical potential μ_L_ = *E*_F_ + *V*_b_/2 and μ_R_ = *E*_F_ – *V*_b_/2 shifted respectively up or down relative to the Fermi energy *E*_F_.^24,61,62,69,70^

It is noted that semilocal functionals can overestimate charge transport in carbon-based systems due to their limited treatment of exchange and correlation effects.^71–73^ In the present work, we have used the vdW-DF functional,^52,67,68^ which augments a semilocal exchange-correlation functional with a nonlocal correlation term to account for van der Waals interactions. While this improves the description of dispersion forces, it does not include exact exchange and may still share some of the limitations observed in semilocal functionals with regard to electronic transport properties. We note this as a limitation of the current approach.

## 3 Results and Discussion

### 3.1 Pristine Devices

We examine prGNR and two types of structural defects: (i) swGNR and (ii) dvGNR. The prGNR, characterized by sp^2^ C–C hybridization, is shown in Figure 1a. The sw defect, a common occurrence in sp^2^ C-based materials, involves a 90° rotation of a C–C bond.^16,17^ This topological transformation leads to the formation of two heptagons connected by two pentagons, as shown in Figure 1c. Conversely, the reconstruction associated with the divacancy defect (585 defects)^19^ results in the formation of two pentagons and one octagon, as shown in Figure 1b. The presence of these two structural defects has been experimentally observed in graphene sheets through STM and SEM images.^74^ Their influence on the structural, electronic, and quantum transport properties, particularly in the presence of biomolecules, warrants detailed investigation. The edges of all three proposed nanostructures are terminated with hydrogen (H) to create a perfect semiconducting nanochannel device. This preparation allows the structures to potentially function as field-effect transistor bioelectronic devices for DNA nucleobase detection.

**Figure 1:**
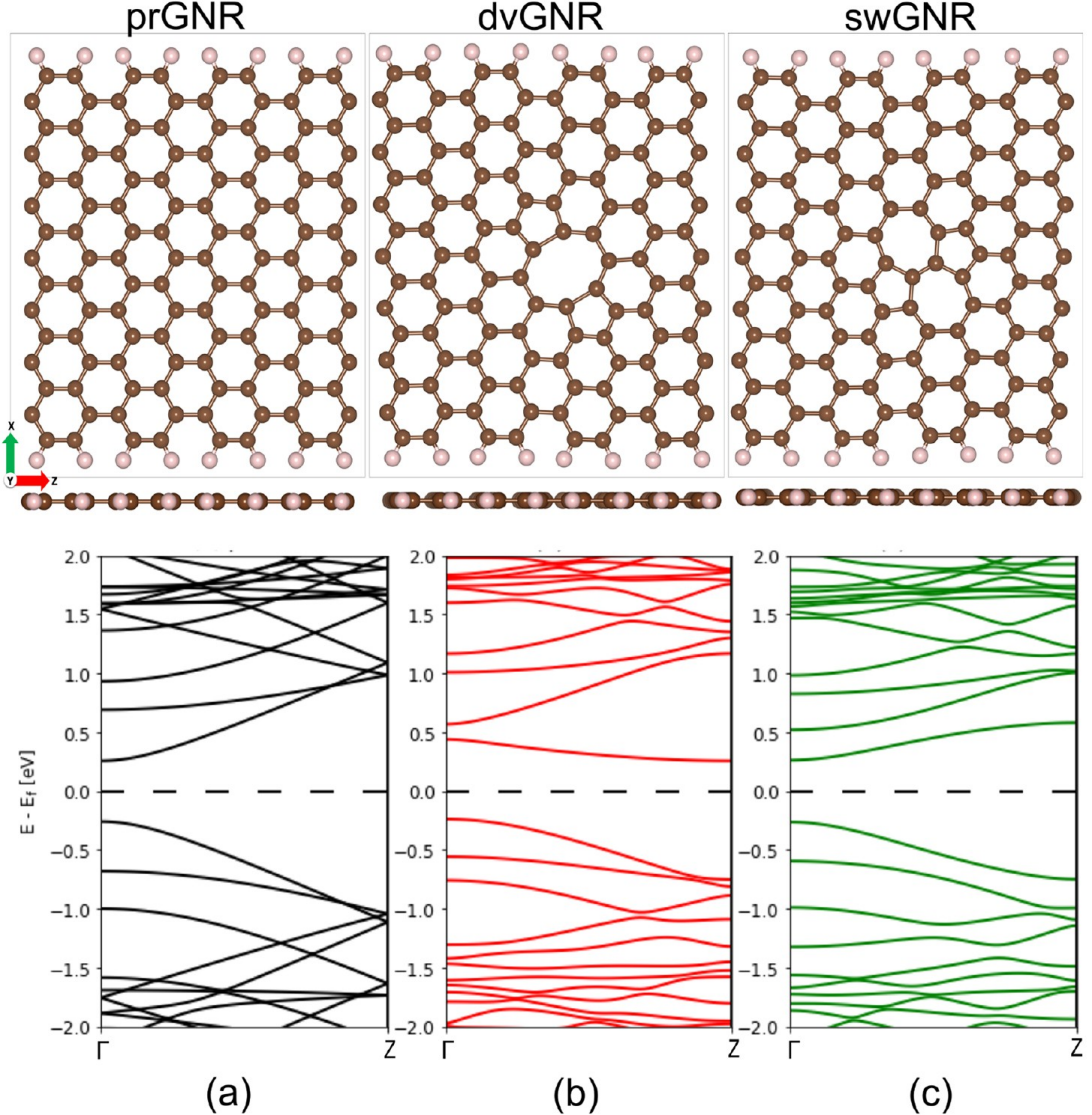
Atomic structures (top view and side view) and electronic band structures of the three modeled supercells: (a) prGNR, (b) dvGNR, and (c) swGNR. The Fermi energy is aligned to zero.

First, we perform structural relaxation of the supercells containing pristine GNR and defects with identical supercell sizes. For this, we use an armchair prGNR (width 15) with H-passivated edges, as shown in Figure 1a. To account for sw- and dv-defects, we employ the supercell model shown in Figure 1b-c. For simplicity, we use the following abbreviations: prGNR (pristine 15 armchair graphene nanoribbon), dvGNR (divacancy 15 armchair graphene nanoribbon), and swGNR (Stone–Wales 15 armchair graphene nanoribbon). In the case of prGNR, the fully relaxed supercell has a width of 17.2 Å (along the zigzag direction) and a length of 17.2 Å (along the armchair direction). For dvGNR, the optimized dimensions are a width of 17.2 Å and a length of 17.0 Å. Similarly, for swGNR, the optimized width is 17.2 Å and the length is 17.3 Å. The sw-defect in GNR does not induce significant structural distortion in the neighboring hexagon rings, whereas the 585 dv-defect causes a more pronounced distortion, particularly along the vertical axis aligned with the octagon.

In Figure 1 (lower panel), we investigate the electronic properties of prGNR and the defective structures (dvGNR and swGNR) by analyzing their respective band structures. The computed electronic band gaps are 0.51 eV (prGNR), 0.49 eV (dvGNR), and 0.52 eV (swGNR). Notably, prGNR and swGNR exhibit a direct band gap semiconducting nature, while dvGNR displays a transition from a direct to an indirect band gap semiconducting nature.

### 3.2 Physisorption of DNA Nucleobases on Nanoribbon Devices

We first performed geometry optimization calculations for prGNR, dvGNR, and swGNR with all four DNA nucleobases positioned above the nanoribbon surfaces. Three distinct configurations–labeled C1 (hollow), C2 (top), and C3 (bridge)–were considered for prGNR, dvGNR, and swGNR, as presented in Figure S2 (Supporting Information). For swGNR, an additional configuration, termed C4 (center and bridge), was explored. Initially, geometries were optimized using the C1 configuration as a reference. Subsequently, based on the optimized C1 geometry, the C2, C3, and C4 configurations were generated, and their relative energies were calculated to identify the most energetically favorable configurations.

The computed relative energy values for each configuration are listed in Table S1 (Supporting Information). As shown, most prGNR and dvGNR systems tend to stabilize in either the C1 or C2 configurations, whereas swGNR systems consistently favor the C1 configuration. These results allow a direct comparison of the relative stability of C2, C3, and C4 configurations, enabling the selection of the most energetically favorable configurations for further study. The most stable configurations of DNA nucleobases adsorbed on prGNR, dvGNR, and swGNR are shown in Figure 2. The calculated binding energies and adsorption heights (*h* in Å) for each nucleobase on different GNR surfaces using various functionals, are listed in Table 1, where *h* denotes the shortest vertical distance between the substrate and the nucleobase. The binding energy (*E*_b_) between target nucleobase (A/G/T/C) and surface (prGNR/dvGNR/swGNR), was calculated as the difference between the total energy of the complex (nucleobase + GNR) and the total energies of systems (nucleobase and GNR surface) individually, as given by (eq 3)­

3 $$E_{b}=E_{\mathrm{GNR}+\mathrm{nucleobase}}-E_{\mathrm{GNR}}-E_{\mathrm{nucleobase}}$$

**Figure 2:**
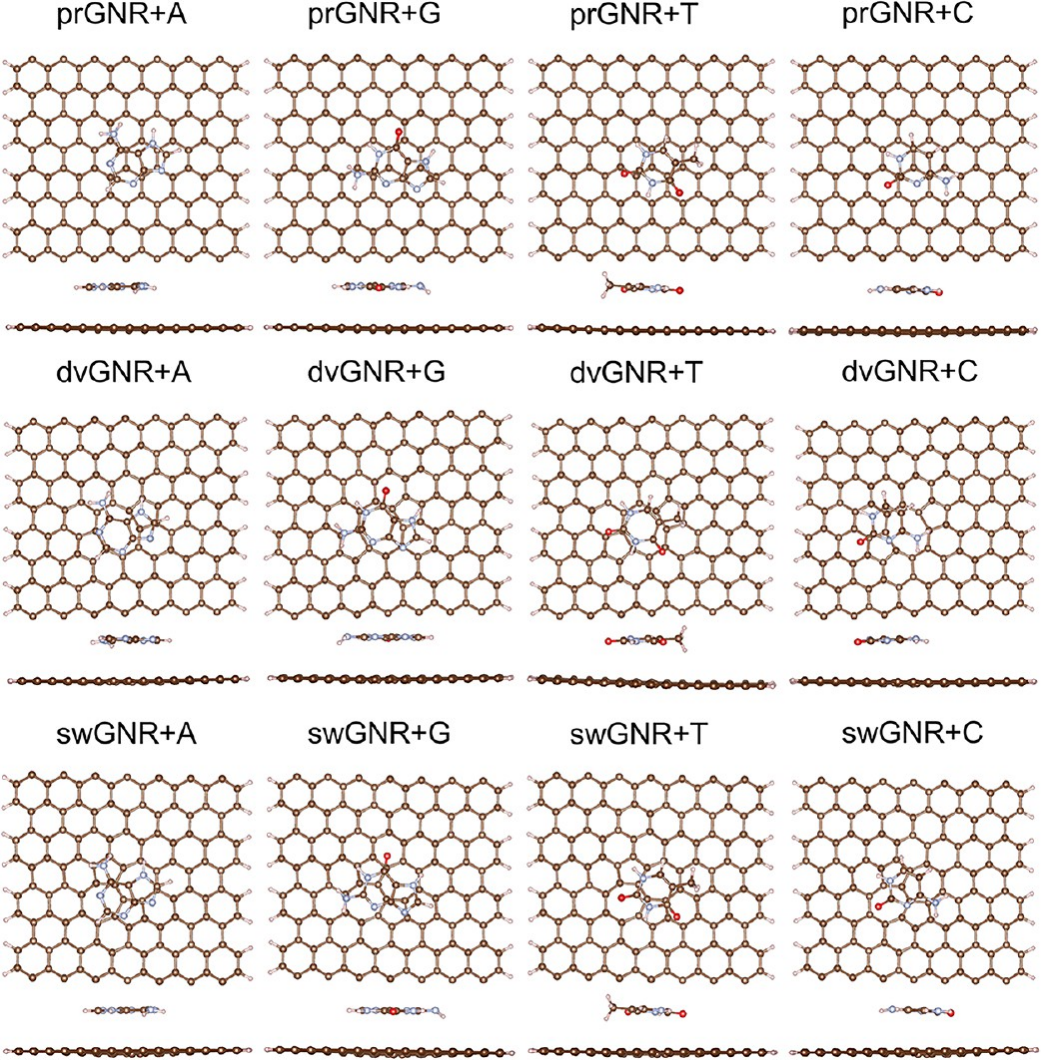
Top and side views of the most energetically stable configurations of adsorbed adenine (A), guanine (G), thymine (T), and cytosine (C) on prGNR, dvGNR, and swGNR substrates are illustrated. In the visualizations, the atoms are color-coded as follows: O (red), N (light blue), C (brown), and H (pink).

**Table 1:** Binding Energies (*E*_**b**_ in eV) and Vertical Adsorption Heights (*h* in Å) of All Four Nucleobases Adsorbed on prGNR, dvGNR, and swGNR Nanodevices^a^

		A		G		T		C	
system	methods	*E*_b_	*h*	*E*_b_	*h*	*E*_b_	*h*	*E*_b_	*h*
prGNR	PBE	–0.09	3.39	–0.10	3.51	–0.06	3.14	–0.08	3.79
	PBE-D2	–0.71	2.84	–0.81	2.67	–0.62	2.65	–0.60	3.01
	vdW-DF2	–0.72	3.06	–0.78	3.04	–0.65	2.88	–0.62	3.30
dvGNR	PBE	–0.10	3.24	–0.09	3.50	–0.07	3.33	–0.09	3.72
	PBE-D2	–0.68	2.73	–0.69	2.79	–0.56	2.64	–0.55	3.01
	vdW-DF2	–0.69	3.01	–0.73	3.03	–0.62	2.78	–0.59	3.30
swGNR	PBE	–0.09	3.27	–0.10	3.34	–0.06	3.36	–0.08	3.71
	PBE-D2	–0.71	2.81	–0.75	2.80	–0.62	2.66	–0.61	3.02
	vdW-DF2	–0.71	3.05	–0.77	3.02	–0.64	2.86	–0.60	3.28

a The values are obtained by using PBE, PBE-D2, and vdW-DF2 methods.

Negative values of binding energies indicate strong interactions. Abbreviations are used to denote systems involving DNA nucleobases, such as prGNR + A (prGNR with Adenine), prGNR + G (prGNR with Guanine), prGNR + T (prGNR with Thymine), and prGNR + C (prGNR with Cytosine). A similar nomenclature is used for dvGNR and swGNR. The binding energies and vertical adsorption heights of the four DNA nucleobases–adenine (A), guanine (G), thymine (T), and cytosine (C)—adsorbed on three types of graphene nanoribbon (GNR) devices (prGNR, dvGNR, and swGNR) were systematically investigated using three computational methods: PBE, PBE-D2, and vdW-DF2. The results (see Table 1) reveal notable differences in interaction strengths and adsorption heights depending on the method used.

The PBE method, which does not explicitly include van der Waals interactions, predicts relatively weak binding energies across all systems, ranging from −0.06 to −0.10 eV. These weak interactions are accompanied by relatively large adsorption heights, between 3.14 and 3.79 Å, indicating a less effective interaction between the nucleobases and the nanoribbon surfaces. The larger distances reflect the PBE method’s inability to accurately capture the π–π stacking interactions that are critical for nucleobase adsorption on graphene-based materials. Similar underestimations have been reported previously for nucleobase adsorption on graphene.^75^ In contrast, the PBE-D2 method, which includes empirical dispersion corrections to account for van der Waals forces, significantly increases the calculated binding energies, with values ranging from −0.55 to −0.80 eV. The corresponding adsorption heights are shorter, between 2.64 and 3.01 Å, indicating relatively stronger interactions. However, in some cases, reduced distances suggest that PBE-D2 may overestimate dispersion effects, leading to overbinding.^76^ This overestimation likely stems from the method’s tendency to exaggerate short-range interactions beyond physically realistic levels.^50,51^ Interestingly, while the binding energies predicted by PBE-D2 are very similar to those obtained with vdW-DF2, the adsorption distances differ slightly. This suggests that while both methods capture the interaction strengths comparably, they describe the interaction geometries with varying degrees of accuracy.

### 3.3 Electronic Bandstructure and Density of States Analysis

The analysis of the binding energies and their impact on the electronic properties of nucleobases adsorbed on prGNR, dvGNR, and swGNR surfaces provides valuable insights into the interaction mechanisms and their influence on device performance. Using the vdW-DF2 functional, which is well-suited for capturing van der Waals interactions, the study reveals how the adsorption of nucleobases affects the Highest Occupied Molecular Orbital (HOMO), Lowest Unoccupied Molecular Orbital (LUMO), and the energy gap (*E*_gap_) of the GNR’s, nucleobases, and combined systems. The calculated values are summarized in Table 2, and the corresponding band structures are presented in Figure 3. In addition to band structure analysis, we also investigated the electronic density of states (DOS) as shown in Figure S3 (Supporting Information), which offers a more detailed understanding of how adsorption redistributes electronic states across different energies. Comparing the DOS of pristine and nucleobase-adsorbed systems reveals clear peak shifts and the appearance of new states, arising from interactions between the target nucleobase and the GNR surface. Notable changes occur when molecular orbitals contribute to the GNR electronic states, particularly in the ranges −4 to −2 eV (occupied states) and 0.5 to 2 eV (unoccupied states), confirmed by electronic DOS (Figure S3) and bandstructure analysis (Figure 3). The decomposed DOS–separating substrate and nucleobase contributions–shows distinct features corresponding to the nucleobase HOMO and LUMO levels, in agreement with the HOMO–LUMO gaps listed in Table 2. Importantly, the total DOS of the adsorbed systems differs from that of the bare substrates, confirming that adsorption induces noticeable changes in the electronic DOS. These changes are due to charge transfer, orbital hybridization, and open new pathways for quantum transport.

**Figure 3:**
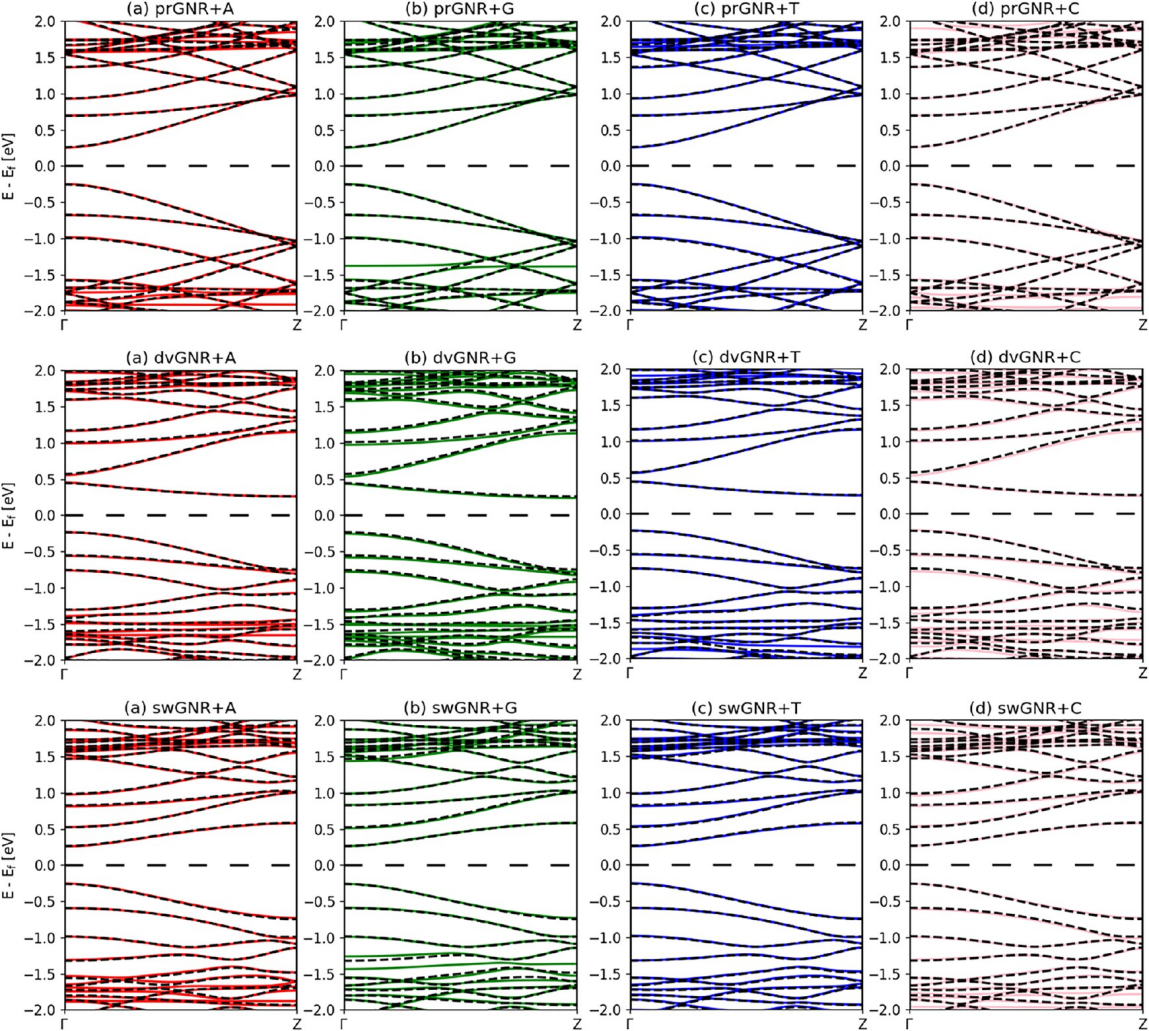
Calculated electronic band structures of adsorbed adenine (A), guanine (G), thymine (T), and cytosine (C) on the prGNR, dvGNR, and swGNR substrates are presented. The band dispersions are plotted along the symmetry lines of the Brillouin zone of the supercell, with the energy zero representing the Fermi level. The black lines depict the band structure of the isolated prGNR, dvGNR, and swGNR substrates, respectively. The vdW-DF2 method was used.

**Table 2:** Calculated HOMO (*E*_H_), LUMO (*E*_L_), and Energy Gap (*E*_gap_) Values for Isolated Nucleobases and GNR’s + Nucleobase Complexes Using the vdW-DF2 Functional^a^

	molecular orbital energies				GNR’s + nucleobase			
nucleobase	*E*_H_	*E*_L_	*E*_gap_	system	*E*_VB_	*E*_CB_	*E*_gap_	Δ*Q* (e)
				prGNR	–3.183	–2.669	0.514	
				prGNR + A	–3.088	–2.585	0.503	–0.005
				prGNR + G	–3.064	–2.562	0.502	–0.009
				prGNR + T	–3.078	–2.575	0.503	–0.019
				prGNR + C	–3.085	–2.582	0.503	0.001
				dvGNR	–3.328	–2.834	0.494	
A	–5.957	–2.130	3.827	dvGNR + A	–3.236	–2.741	0.495	–0.004
G	–5.494	–2.051	3.443	dvGNR + G	–3.223	–2.716	0.507	–0.010
T	–6.407	–2.631	3.776	dvGNR + T	–3.223	–2.736	0.487	–0.016
C	–5.996	–2.330	3.666	dvGNR + C	–3.216	–2.745	0.471	0.001
				swGNR	–3.234	–2.709	0.525	
				swGNR + A	–3.137	–2.623	0.514	–0.003
				swGNR + G	–3.116	–2.595	0.521	–0.009
				swGNR + T	–3.131	–2.617	0.514	–0.019
				swGNR + C	–3.136	–2.609	0.527	0.001

a The table presents the molecular orbital energies (in eV) and energy gap values for both the isolated nucleobases and the corresponding complexes formed with prGNR, dvGNR, and swGNR. Here, E_VB_ and E_CB_ represent the valence band maximum and conduction band minimum. The computed charge transfer Δ*Q* (e) values of the GNR surface after the adsorption of a target nucleobase.

From Table 2, the computed HOMO levels of isolated A, G, T, and C nucleobases range from −5.49 to −6.41 eV, reflecting their ionization energies and relative electron-donating strengths. Guanine, with the highest (least negative) HOMO at −5.49 eV, is the most easily ionized, suggesting stronger interaction with GNR surfaces among other nucleobases. The effect of adsorption on *E*_gap_ depends strongly on the substrate type. For the prGNR substrate, all nucleobases reduce *E*_gap_ by approximately 0.01 eV, indicating a uniform and stable interaction environment where conduction and valence bands are similarly stabilized. For the dvGNR substrate, divacancy defects cause more variable *E*_gap_ changes, consistent with stronger binding energies. Guanine increases the gap by 0.01 eV, while cytosine decreases it by 0.02 eV. These variations stem from the diverse local electronic environments created by defects, which enhance π–π interactions (hybridization between molecule and substrate), electronic DOS, and differentially stabilize molecular orbitals. In the swGNR substrate, adsorption effects are smaller but still varied; for example, cytosine uniquely increases *E*_gap_ by 0.01 eV, likely due to specific interactions with Stone–Wales defects. Among all the substrates, dvGNR shows the most pronounced defect-induced modifications, primarily due to 585-type vacancies and the associated structural distortions that strengthen electrostatic interactions and orbital hybridization between nucleobases and defected GNR π-orbitals. These stronger couplings lead to significant changes in both DOS and band structure, underscoring the importance of defect engineering in tailoring GNR-based molecular sensing devices for optimal performance.

### 3.4 Charge Density and Charge Transfer Analysis

The computed binding energy values and the corresponding changes in energy levels and the energy gap indicate that our vdW-DF2 functional effectively captures the π–π interactions between GNR substrates and nucleobases. To further elucidate the nature of the interaction between the target nucleobases and the GNR surfaces, we examine the charge density difference (Δ_ρ_) plots and the Bader charge transfer (CT) values by using the vdW-DF2 method. This analysis highlights the rearrangement of charge at the GNR+nucleobase interface, calculated by using eq (eq 4)­

4 $$\Delta_{\rho }=\rho_{\mathrm{GNR}+\mathrm{nucleobase}}-\rho_{\mathrm{GNR}}-\rho_{\mathrm{nucleobase}}$$

here, ρ_GNR+nucleobase_, ρ_GNR_, and ρ_nucleobase_ represent the charge densities of the combined GNR + nucleobase system (with GNR being prGNR, dvGNR, or swGNR, and the nucleobases being A, G, T, or C) and its isolated components–namely the GNR substrate and the nucleobase molecules, respectively.

In Figure 4, we present the Δ_ρ_ for A, G, T, and C adsorbed on prGNR, dvGNR, and swGNR substrates. The lobes in the Δ_ρ_ plots indicate regions of electron depletion (cyan) and electron accumulation (yellow). These visualizations reveal that charge rearrangement occurs at the interface in all four GNR+nucleobase systems. This rearrangement induces an interfacial dipole, leading to shifts in the electronic states.

**Figure 4:**
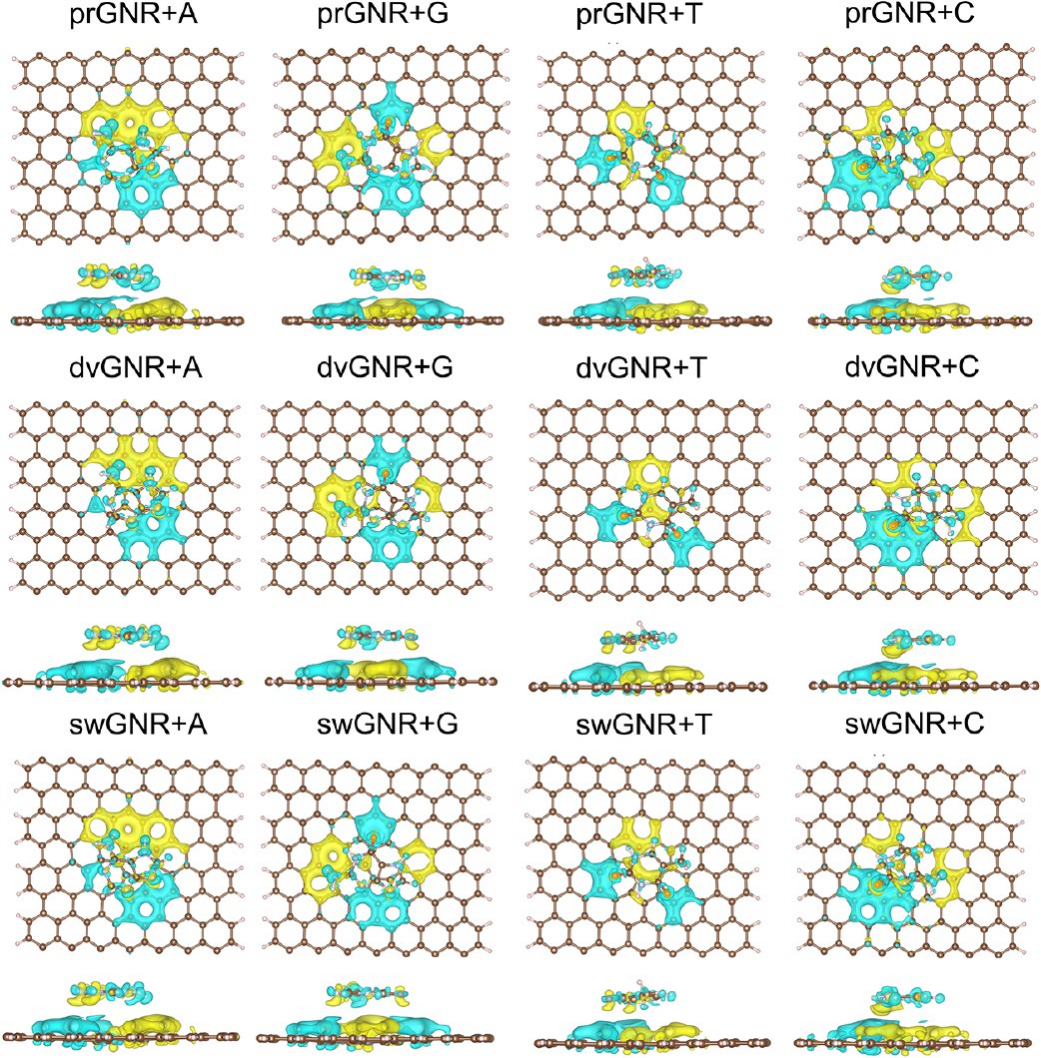
Calculated charge density difference plots, Δ_ρ_, are presented for adsorbed A, G, T, and C on prGNR, dvGNR, and swGNR substrates, with an iso-surface value of 0.005 e/Å^3^. The cyan-colored and yellow-colored iso-surfaces indicate regions of electron depletion and electron accumulation, respectively. The vdW-DF2 method was used.

To gain deeper insight into how charge is distributed between the substrate and the nucleobase, we carried out the Bader charge analysis^77–79^ for the prGNR, dvGNR, and swGNR systems. While the Bader charge partitioning method is not always quantitatively precise, it is valuable for identifying qualitative trends that can reveal important features of these systems. The total charge of the GNR substrate was obtained by summing the Bader charges of only the graphene atoms. The net charge transfer, Δ*Q*, was calculated as (eq 5)­

5 $$\Delta Q=Q_{\mathrm{GNR}+\mathrm{nucleobase}}-Q_{\mathrm{isolated}‐\mathrm{GNR}}$$

where *Q*_GNR+Nucleobase_ and *Q*_isolated‑GNR_ represent the total charges of the GNR sheet after and before molecular adsorption, respectively. A negative Δ*Q* indicates electron transfer from the molecule to the GNR (molecule acts as donor), whereas a positive Δ*Q* indicates electron transfer from the GNR to the molecule (molecule acts as acceptor).

As shown in Table 2, the A, G, and T nucleobases transfer electrons to the GNR substrates (negative Δ*Q*), whereas C acts as a weak electron acceptor (small positive Δ*Q*). Among them, thymine shows the largest charge transfer (−0.019 e), followed by guanine, adenine, and cytosine, consistent with the binding energy trends in Table 1. Structural defects in the GNRs (dv and sw types) have only a minor effect on Δ*Q*. These results are also in agreement with the earlier electronic DOS analysis and discussion of *E*_gap_.

### 3.5 Quantum Transport Studies

Electronic transmission responses are valuable for the electronic detection of DNA nucleobases. Therefore, we investigate the quantum transport properties resulting from the interaction of DNA nucleobases with our proposed GNR-based nanochannel devices, specifically prGNR, dvGNR, and swGNR. The nanochannel devices, as shown in Figure S1 (Supporting Information), are designed to calculate the transmission responses at zero bias upon the adsorption of DNA nucleobases (A, G, T, and C).

#### 3.5.1 Pristing GNR Nanochannel Devices

We investigate the quantum transport properties of all three proposed GNR devices. In Figure 5, we show the impact of structural defects on the electronic and quantum transport properties of bare GNR devices. The transmission curves at zero bias (*T*(*E*, *V*_b_ = 0) or *G*_0_) in Figure 5(a) compare the transmission of prGNR, dvGNR, and swGNR as a function of energy relative to the Fermi level. Within an energy range of −2.5 to +2.5 electron volts (eV) around the Fermi level, multiple conducting channels are observed, which can be attributed to states associated with carbon atoms. These states facilitate the quantum transport of electrons through the GNR nanochannel devices. The prGNR device exhibits integer values of transmission spectra, which indicates the presence of discrete conduction channels.^20,22^ However, GNR devices with defects, such as dvGNR and swGNR, show sharp declines in the transmission curve within the specified energy range, as seen in Figure 5(a). This reduction in transmission suggests that the defects induce significant alterations in the intrinsic electron transport properties of the GNR device. The presence of defects, such as dv and sw configurations, leads to the formation of localized electronic states within the GNR structures. These localized states interact with the delocalized states leading to destructive interferences known as *Fano resonance*,^20,22^ which is responsible for the sharp dips in transmission observed in the dvGNR and swGNR devices.

**Figure 5:**
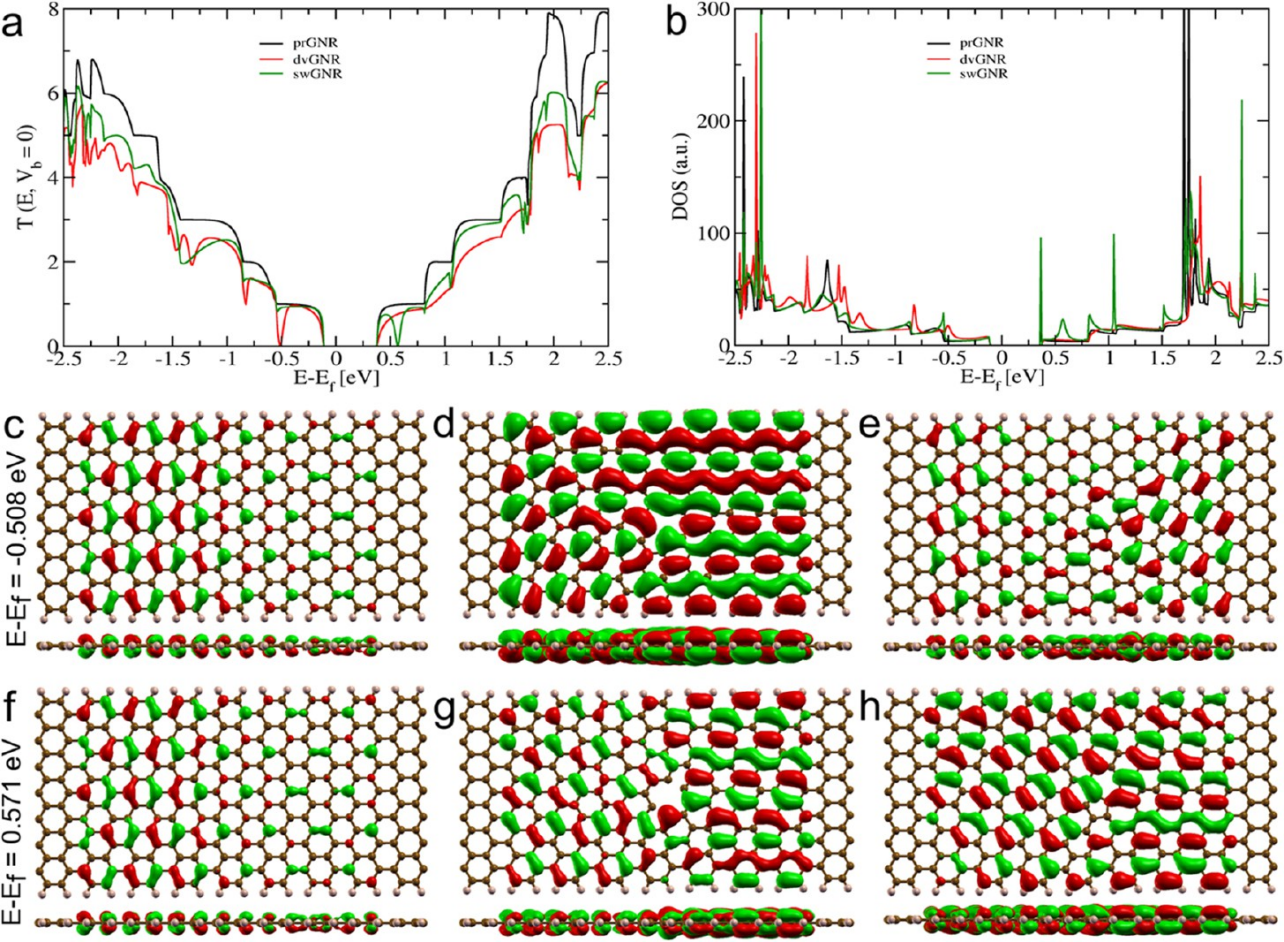
Electronic and quantum transport properties of prGNR, dvGNR, and swGNR devices. (a) Transmission curve (in *G*_0_ at *V*_b_ = 0). (b) The density of states (DOS). MOs of (c, f) prGNR, (d, g) dvGNR, and (e, h) swGNR at energies (*E* – *E*_f_) of −0.508 and 0.571 eV. The sharp dip in the transmission curve of dvGNR and swGNR at different energy values occurs due to the *Fano resonance*.

Furthermore, the specific nature of the defects influences the transmission properties differently. The dvGNR device exhibits several pronounced resonance peaks (sharp drops) in the transmission curve, particularly in the negative energy region. In contrast, the swGNR device induces similar resonance peaks in the positive energy region. These resonance peaks are a direct consequence of the interaction between electron waves and the defect sites, leading to localized resonant states within the energy spectrum of the GNR devices. The density of states (DOS) shown in Figure 5(b) corroborates this by highlighting the introduction of defect-related states, which differ notably from the DOS spectra of prGNR, further influencing the quantum transport characteristics.

The molecular orbitals (MOs) visualized in Figure 5(c–h) at energies −0.508 and −0.571 eV provide additional insight into the impact of these defects. The perturbation of electron density distribution around the defect sites, particularly at the energies corresponding to the *Fano resonance* dips, is evident in these MOs. This perturbation illustrates how defects significantly alter the electronic environment, leading to the observed changes in transmission. The observed correlation between defect-induced changes in MOs, DOS, and transmission spectra underscores the critical role of defects in modulating the electronic and transport properties of GNR devices, which is pivotal for their application in nanoscale electronic devices and biosensing technologies.

Additionally, these findings are crucial for applications such as DNA sequencing, where the sharp drops in transmission could be leveraged for detecting nucleobases. As a single-stranded DNA (ssDNA) passes through a GNR-based nanochannel device, the interaction between the nucleobases and the GNR surface alters the local electronic environment,^22^ potentially shifting the energy levels of the defect-induced localized states. This interaction, particularly in defected GNRs, can result in distinct transmission signatures for each nucleobase, enabling their precise identification through the sharp, defect-induced resonance peaks. Thus, the enhanced sensitivity of defected GNRs due to Fano resonances highlights their potential as highly effective components in next-generation DNA sequencing technologies.

### 3.6 DNA Adsorption-Induced Changes in Quantum Transport of Graphene Nanoribbon Devices

The transmission spectra shown in Figure 6, 7 and 8, demonstrate sharp drops (dips in transmission resonance peaks) upon nucleobase adsorption. This phenomenon arises due to the orbital overlap (i.e, π–π interactions) between the GNR surface and the target nucleobases, leading to sharp dips in the transmission spectra around the Fermi level. Additionally, the position of the resonance dip can be controlled by applying an externally applied gate voltage.^20,22^

**Figure 6:**
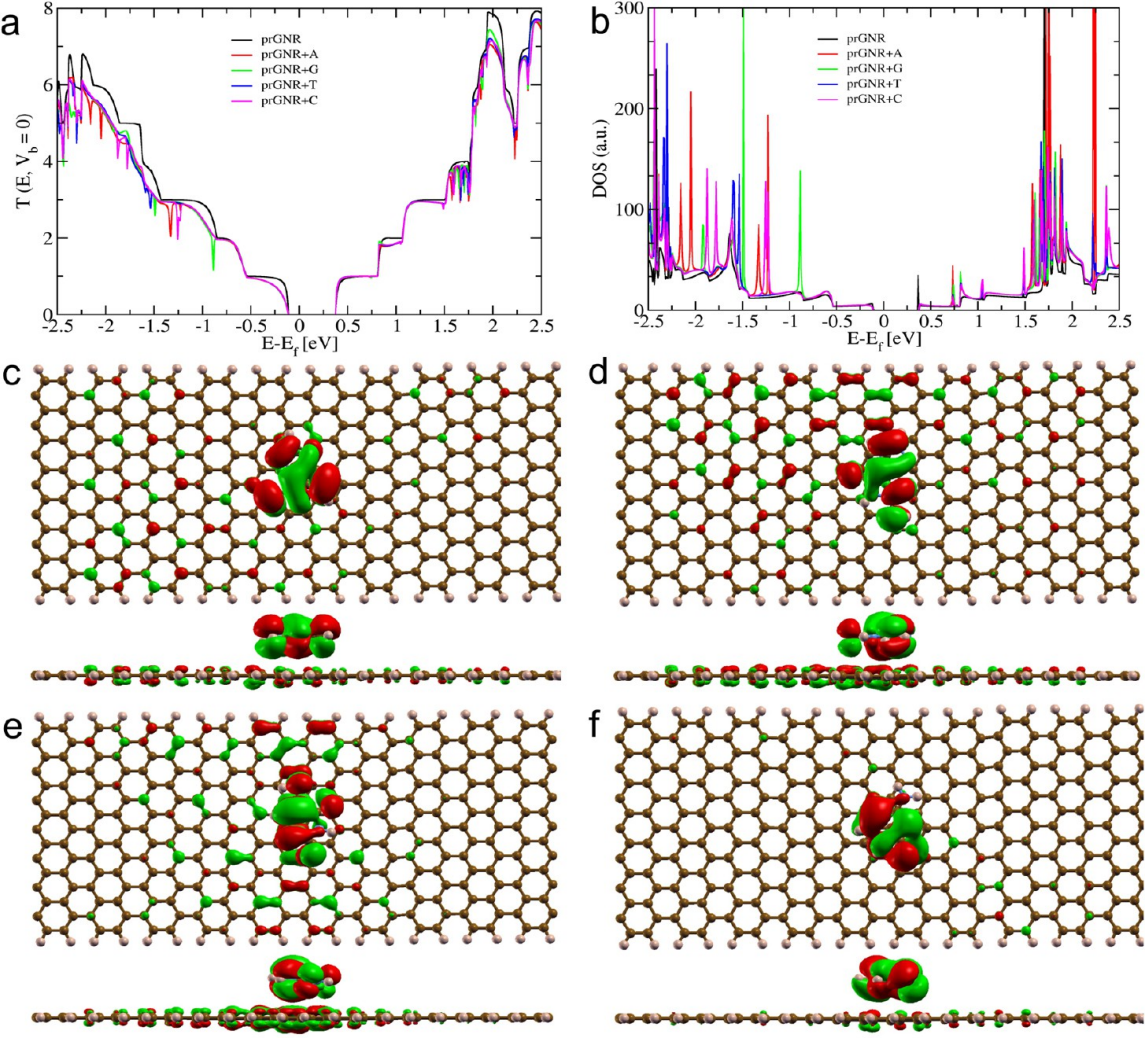
Electronic and quantum transport properties of a prGNR device stacked optimally with DNA nucleobases. (a) Transmission curve (in *G*_0_ at *V*_b_ = 0). (b) The density of states (DOS). (c–f) MOs of a prGNR with stacked (c) Adenine at energy (*E* – *E*_f_) of −1.322 eV, (d) Guanine at energy (*E* – *E*_f_) of −0.888 eV, (e) Thymine at energy (*E* – *E*_f_) of −1.541 eV, and (f) Cytosine at energy (*E* – *E*_f_) of −1.252 eV. The sharp dip in the transmission curve of prGNR + nucleobase at different energy values occurs due to the *Fano resonance* between its MO and prGNR.

**Figure 7:**
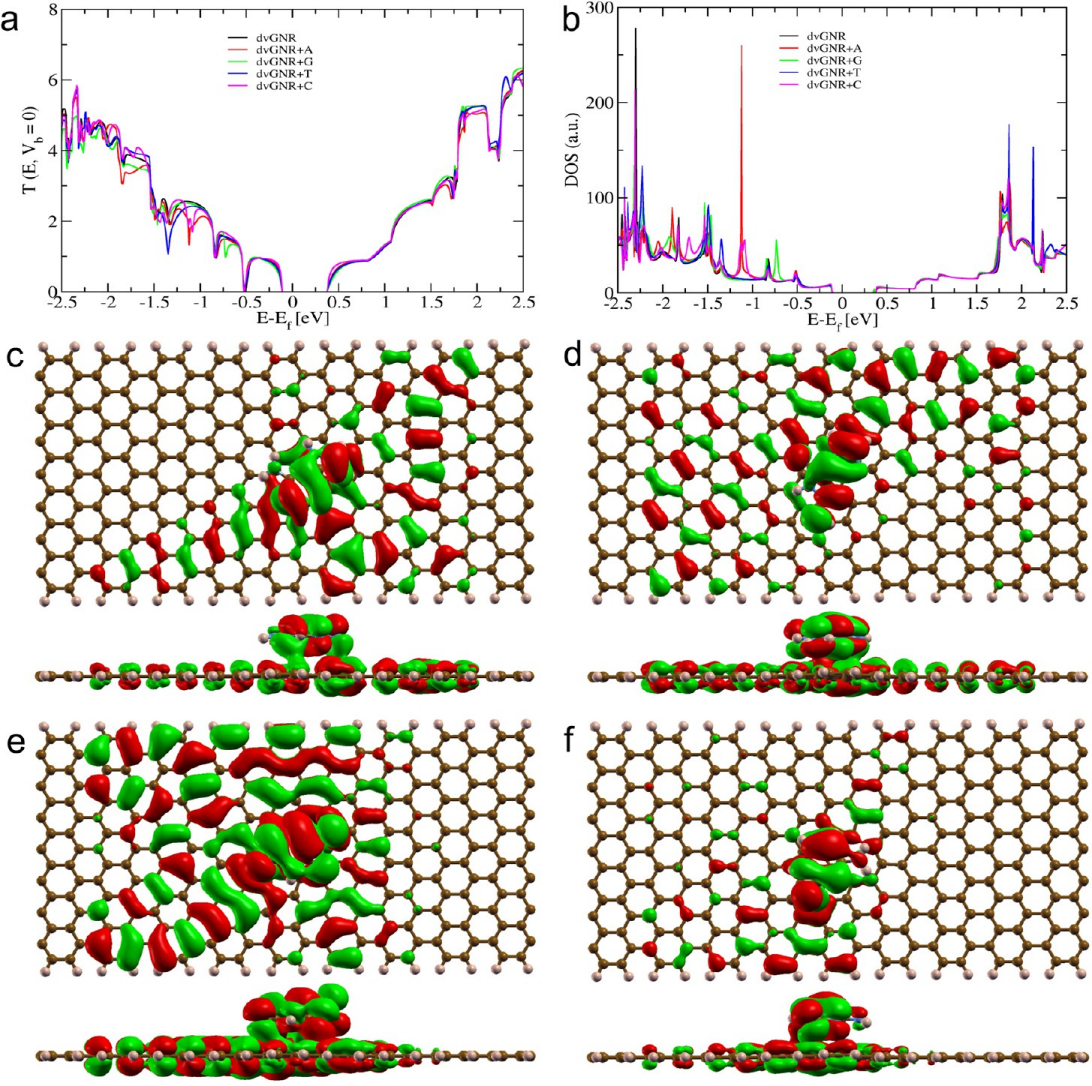
Electronic and quantum transport properties of a dvGNR device stacked optimally with DNA nucleobases. (a) Transmission curve (in *G*_0_ at *V*_b_ = 0). (b) The density of states (DOS). (c–f) MOs of a dvGNR with stacked (c) Adenine at energy (*E* – *E*_f_) of −1.843 eV, (d) Guanine at energy (*E* – *E*_f_) of −0.725 eV, (e) Thymine at energy (*E* – *E*_f_) of −1.345 eV, and (f) Cytosine at energy (*E* – *E*_f_) of −1.086 eV. The sharp dip in the transmission curve of dvGNR + nucleobase at different energy values occurs due to the *Fano resonance* between its MO and dvGNR.

**Figure 8:**
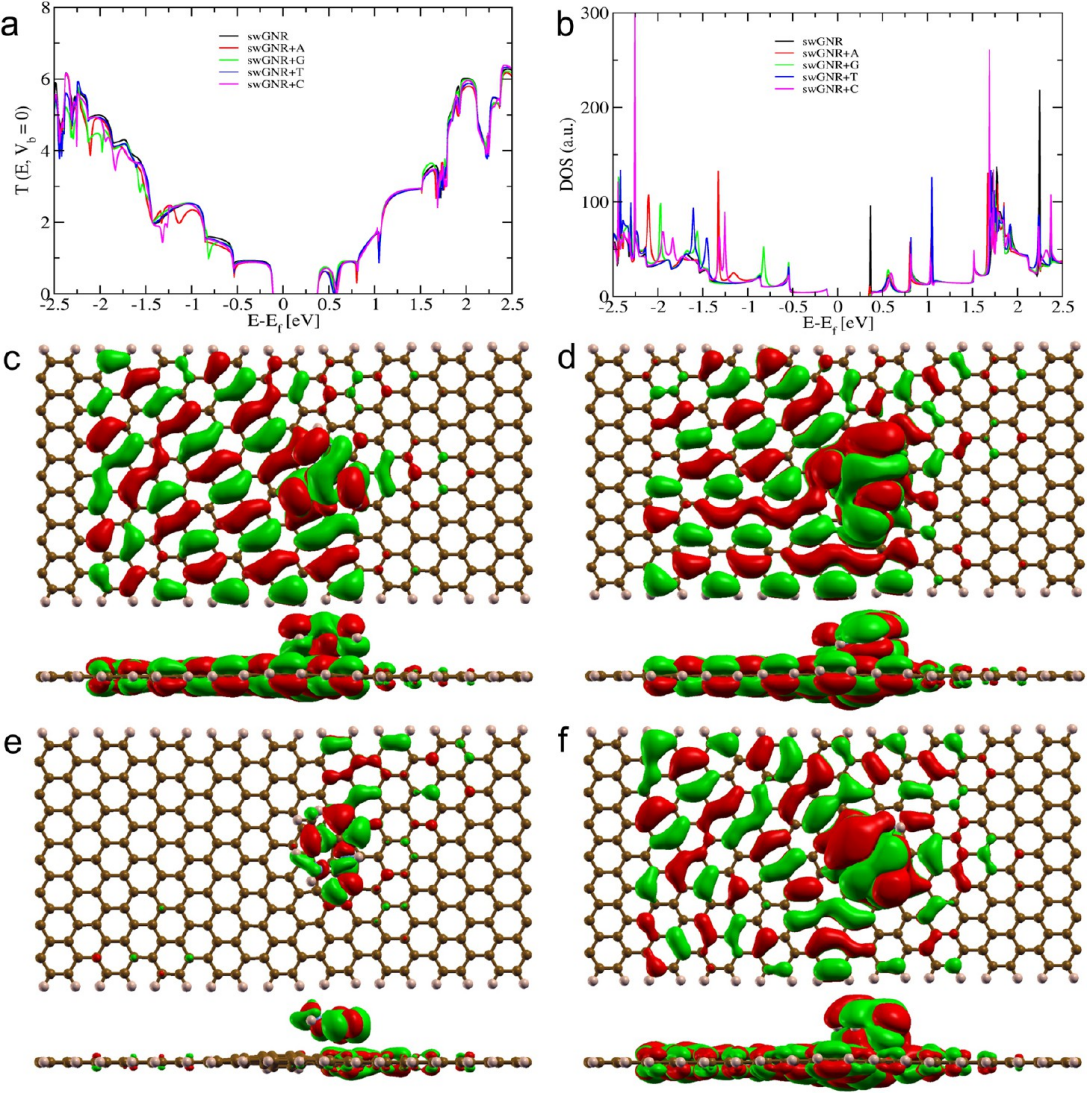
Electronic and quantum transport properties of a swGNR device stacked optimally with DNA nucleobases. (a) Transmission curve (in *G*_0_ at *V*_b_ = 0). (b) The density of states (DOS). (c–f) MOs of a swGNR with stacked (c) Adenine at energy (*E* – *E*_f_) of −1.138 eV, (d) Guanine at energy (*E* – *E*_f_) of −0.815 eV, (e) Thymine at energy (*E* – *E*_f_) of −1.601 eV, and (f) Cytosine at energy (*E* – *E*_f_) of −1.264 eV. The sharp dip in the transmission curve of swGNR + nucleobase at different energy values occurs due to the *Fano resonance* between its MO and swGNR.

#### 3.6.1 Interaction Analysis Between prGNR and Nucleobases

In the transmission spectra (Figure 6) of prGNR+nucleobase systems, each nucleobase (A, G, T, and C) exhibits nearly symmetric resonance dips at different energy values. These resonance dips occur both below and above the Fermi level, indicating significant changes in transmission properties upon nucleobase adsorption, thereby underscoring their potential for electronic detection. For instance, adenine stacked with prGNR shows a pronounced dip around −1.322 eV in both the transmission spectra and DOS curves (Figure 6(a,b)). This dip arises from strong *Fano resonance* due to the interaction between prGNR and adenine, obstructing the ballistic transmission channels of the prGNR device, as confirmed by plotting the responsible molecular orbital (MO) (Figure 6c). The MO at the dip position corresponds to the HOMO and is primarily localized on the adenine molecule with partial contribution from the prGNR device. Similarly, guanine exhibits a dip at around −0.888 eV (Figure 6a), with a mixed MO at the dip position representing the HOMO, as shown in Figure 6d. Cytosine also shows a dip at around −1.252 eV, with the HOMO strongly localized on cytosine contributing to the resonance dip (Figure 6f).

In contrast, thymine nucleobase exhibits a dip position around −1.541 eV, which is relatively farther compared to the other three nucleobases. This discrepancy is attributed to the position of the HOMO, which is strongly localized on the molecule with distributed density on the prGNR device (Figure 6e). The interaction between the p_*x*_ and p_*y*_ orbitals generates a π–π interaction,^20,22^ responsible for the observed *Fano resonance*. Therefore, coupling the prGNR device with the target molecule leads to a sharp drop in the transmission spectra, known as *Fano resonance*. These Fano resonance peaks have the potential for nucleobase detection due to their unique resonance peak position and molecular orbital characteristics.

#### 3.6.2 Interaction Analysis Between dvGNR and Nucleobases

Further, we investigated our modeled defected dvGNR device interacting with target DNA nucleobases. The calculated transmission spectra, DOS, and MOs are presented in Figure 7. Similar to prGNR, each target nucleobase stacked on the dvGNR nanodevice induces a sharp drop in the transmission spectra (Figure 7a). However, the overall transmission values are smaller compared to the prGNR results. The observed resonance dips correlate well with the DOS curve, occurring both below and above the Fermi level. Notably, the dvGNR device exhibits strong π–π channels throughout its structure, showing robust coupling with the target nucleobases which correlates well with the calculated stronger binding energy and energy gap values. For instance, adenine stacked with the dvGNR exhibits a prominent dip at around −1.843 eV in both the transmission spectra and DOS (Figure 7a,b). This pronounced dip confirms *Fano resonance* from the interaction between dvGNR and adenine. MO responsible for this specific resonance dip is shown in Figure 7c, with strong electron delocalization on both the adenine and dvGNR surface, facilitating π–π interaction and impeding the ballistic transmission channels of the dvGNR. A similar behavior was observed for thymine nucleobase (Figure 7e), with a dip at around −1.345 eV, indicating a preference for coupling around the defected position. As discussed earlier, dvGNR exhibits strong π–π orbital localization due to dv defects. In contrast, guanine and cytosine block relatively fewer π–π-dvGNR channels. Guanine shows a dip at around −0.725 eV, with a mixed MO at the dip position (Figure 7d). Cytosine nucleobase exhibits a dip at around −1.086 eV, with the MO localized on both cytosine and dvGNR channels (Figure 7f) contributing to the resonance dip.

#### 3.6.3 Interaction Analysis Between swGNR and Nucleobases

In our final system, the swGNR device interacts with target DNA nucleobases, resulting in a complex interplay of quantum transport and electronic properties. The calculated transmission spectra, DOS, and MOs are shown in Figure 8. This figure shows that each nucleobase induces distinctive resonance dips in transmission spectra, occurring at specific energy levels: adenine at −1.138 eV, guanine at −0.815 eV, cytosine at −1.601 eV, and thymine at −1.264 eV. These dips, aligned with the DOS curve, highlight the significant influence of nucleobase adsorption on the electronic behavior of the swGNR device. The swGNR device exhibits robust π–π channels, fostering strong coupling with target molecules. Notably, the occurrence of *Fano resonance*, exemplified by sharp dips in transmission functions, reveals intricate π–π interactions between swGNR and adsorbed nucleobases. This interaction, detailed through MOs (Figure 8c-f), shows significant electron delocalization on both the nucleobase and swGNR surface, altering the transmission landscape of the system.

#### 3.6.4 Conductance Sensitivity Analysis of Nucleobases Using GNR Nanochannel Devices

Although our proposed devices, both with and without defects, are capable of detecting each nucleobase type in a DNA sequence due to their distinct transmission spectras and *Fano resonance* peaks, we focus on calculating the sensitivity for experimental validation. Figure 9 presents a comparative analysis of sensitivity (*S*%) for detecting all four nucleobases—A, G, T, and C—using three types of GNR devices: prGNR, dvGNR, and swGNR. Sensitivity is calculated using the formula:

6 $$S\%=\left|\frac{G-G_{0}}{G_{0}}\right|\times 100\%$$

**Figure 9:**
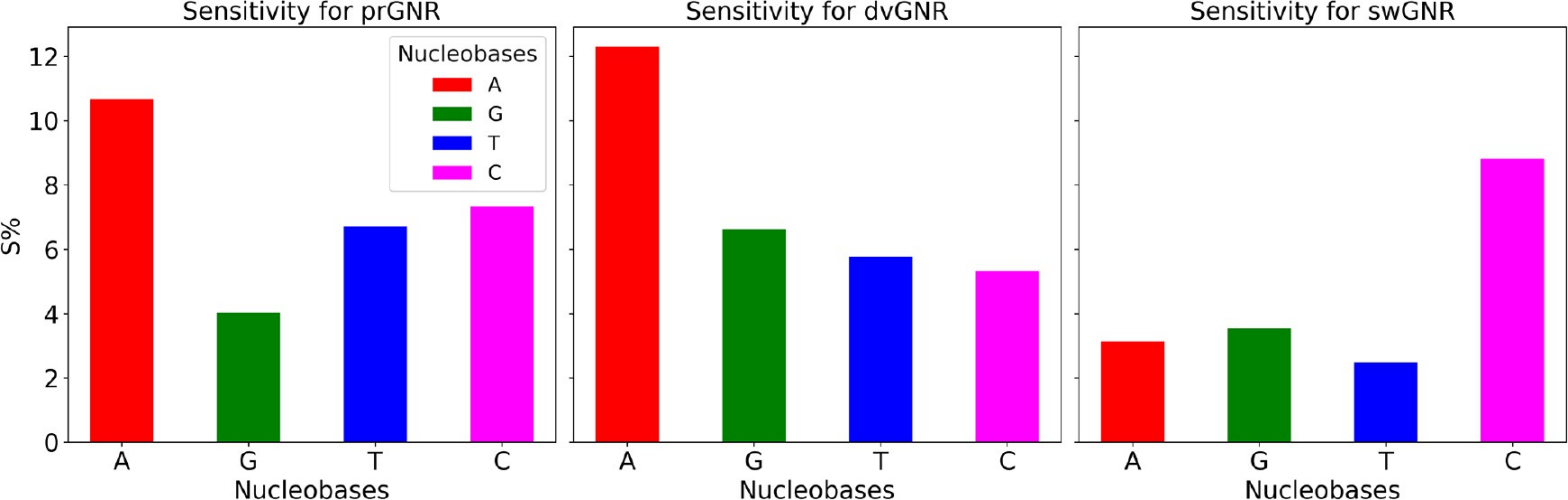
Conductance sensitivity (*S*%) of nucleobases A, G, T, and C detected using prGNR, dvGNR, and swGNR devices. The energy value of −1.80 eV was selected for these measurements, but in practice, this can be adjusted via an external gate voltage to align with the device’s Fermi level.

where *G* represents the conductance of the GNR device with a nucleobase, and *G*_0_ is the reference conductance of the GNR device without any nucleobase. This calculation provides insight into how effective each GNR device can distinguish between different nucleobases based on their interaction with the device.

The conductance sensitivity was calculated from Figures 6–8 at an energy value of 1.80 eV for each nucleobase interacting with various GNR nanochannel devices, as shown in Figure 9. Although the energy value of −1.80 eV was selected for these measurements, it can be adjusted via an external gate voltage to align with the device’s Fermi level. Other energy values may also be selected for detection, considering that graphene devices begin to rupture after 3.0 V of applied bias.^80^

The results indicate that prGNR exhibits the highest sensitivity values for most nucleobases compared to dvGNR and swGNR. This superior performance is attributed to prGNR’s distinct electronic characteristics, which result in more pronounced changes in transmission when nucleobases interact with the device. The unique defect-free configurations, electronic structure, and transmission spectra of prGNR enhance its ability to detect subtle variations in nucleobase presence, making it a more effective choice for nucleobase detection in practical applications. The *S*% values for A, G, T, and C when interacting with the prGNR nanochannel device are approximately 10.67%, 4.03%, 6.71%, and 7.34%, respectively. In contrast, dvGNR shows sensitivity values of 12.30% (A), 6.63% (G), 5.76% (T), and 5.32% (C), while swGNR exhibits lower sensitivity values of 3.13% (A), 3.53% (G), 2.48% (T), and 8.82% (C). The lower sensitivity observed in dvGNR and swGNR, except for dvGNR with A and swGNR with C, is likely due to the nature of their structural defects and reduced transmission spectra. dvGNR, with its specific defect-induced resonance peaks, and swGNR, with its complex interaction patterns, do not achieve the same level of sensitivity as prGNR. Therefore, prGNR is identified as the most promising device for detecting nucleobases, thanks to its superior sensitivity and the ability to leverage *Fano resonances* for precise electronic detection.

#### 3.6.5 Current–Voltage Response and Sensitivity Analysis of Nucleobases Using GNR Nanochannel Devices

To understand the impact of externally applied bias voltages on prGNR, dvGNR, and swGNR devices with and without target nucleobases, we calculated the current–voltage (*I*–*V*) response profiles using the Non-Equilibrium Green’s Function (NEGF) approach, as discussed above in computation details section. Bias voltages were applied up to 1.8 V for the prGNR devices, and up to 0.8 V for both dvGNR and swGNR devices, with and without nucleobases. In our device, graphene electrodes are employed. Experimental studies have shown that graphene rupture typically occurs at voltages above 3.1 V.^80^ Since the maximum bias applied in our simulations is limited to 1.8 V (prGNR) and 0.8 V (dvGNR/swGNR), these voltages remain well below the reported breakdown threshold. Therefore, we do not anticipate device failure at the operating conditions considered here. To quantify the change in current due to the presence of nucleobases, we calculated the difference in electric current values using the equation below

7 $$\Delta I=I-I_{0}$$

here, *I* is the electric current of the GNR + nucleobase devices, and *I*_0_ is the electric current of the GNR nanochannel devices without nucleobase. The results of this analysis are presented in Figure 10. In Figure 10a–c, we demonstrate that negligible electric current is observed below a 0.5 V bias, attributed to the semiconducting nature of the GNR electrodes with an electronic bandgap of 0.5 eV (see Figure 3 and Table 2). This indicates that a bias greater than 0.5 V is required to observe a current response in these devices. Once the bias exceeds 0.5 V, all devices begin to exhibit an electric current response, both with and without nucleobases.

**Figure 10:**
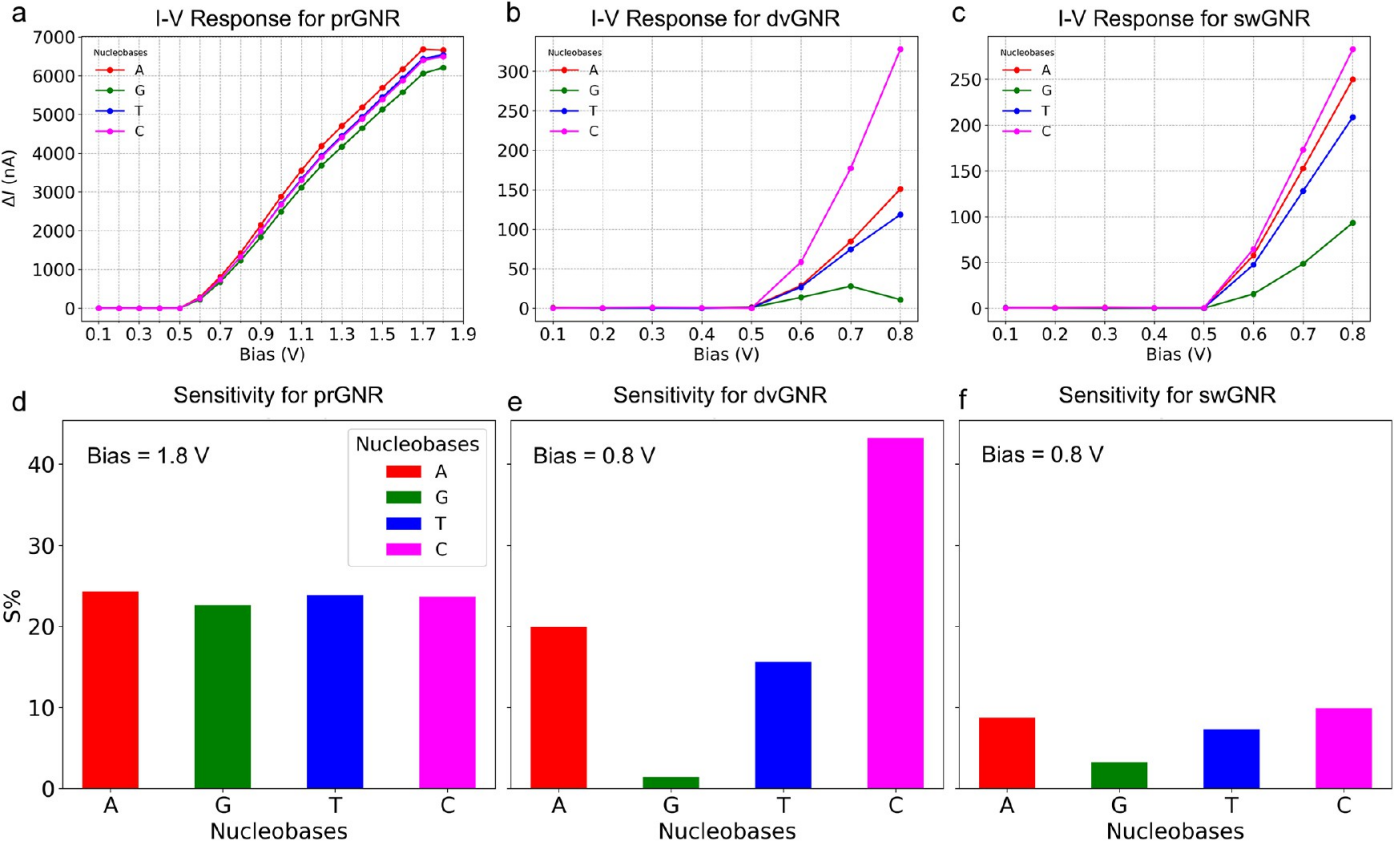
*I*–*V* Response and Sensitivity Analysis: (a–c) Differences in current response for prGNR, dvGNR, and swGNR with nucleobases (A, G, T, C) at biases up to 1.8 and 0.8 V. (d–f) Current sensitivity of prGNR at 1.8 V, showing uniform response across nucleobases. (e) dvGNR sensitivity at 0.8 V, with cytosine (C) showing the highest sensitivity. (f) swGNR sensitivity at 0.8 V, with cytosine (C) showing the highest, but more uniform sensitivity.

For the prGNR device (Figure 10a), the electric current gradually increases as the bias increases from 0.5 to 1.8 V. All nucleobases produce similar electric current trends with only minor differences. However, despite these small variations, the electric current values are significant, in the nanoampere (nA) range, which are detectable experimentally.^81^ In contrast, the dvGNR and swGNR devices (Figure 10b,c) exhibit more pronounced differences in electric current values at an applied bias of 0.8 V. After 0.5 V, the distinct electric current profiles of these devices make it easier to differentiate between nucleobase types. This variation in electric current profiles across the three device types can be attributed to their structural characteristics, electronic properties, transmission spectra, and MOs discussed above.

We also investigated the reason for the apparent decrease in current at an applied bias of 0.8 V for the dvGNR + G device. We found that the current for both the dvGNR and dvGNR + G devices increases steadily up to 0.8 V (see below Figure S4). The apparent drop seen in Figure 10b for the dvGNR + G device at this voltage is not due to a decrease in actual current. Instead, it happens because the difference in current between dvGNR + G and bare dvGNR device is smaller at 0.8 V than it is at 0.7 V. As a result, the differential signal shown in Figure (Figure 10b) appears lower at 0.8 V.

Specifically, defects in GNRs lead to localized electronic states that interact with delocalized states, resulting in prominent *Fano resonances*, which are characterized by sharp dips in the transmission spectra. In defected GNR devices with stacked nucleobases, the transmission curve exhibits sharp drops at the characteristic MO energies of each nucleobase due to the Fano resonance between the localized MOs of the nucleobase and the continuum π-band of the GNR (as seen in Figures 7 and 8). Additionally, under an externally applied bias, the shifting of resonance peak positions plays a crucial role in shaping the electric current profiles. Since the MO states are close to the Fermi level, they can contribute to the electric current as the applied bias increases beyond 0.5 V. The molecular orbital near the Fermi level–most likely the HOMO–is indeed responsible for the transmission that contributes to the current as the bias goes beyond 0.5 V. To analyze this, we also calculated the bias-dependent transmission function of the dvGNR+G system to see how both the transmission and the MO positions change with applied bias (Figure S5, **top panel**). As the bias increases from 0 to 0.5 V and up to 0.8 V, the overall transmission decreases slightly, and the peak shifts closer to the Fermi level. Importantly, the transmission exactly at the Fermi level increases with bias, which helps explain the rise in current. Looking at the molecular orbital plots in Figure S5, **down panel**; the orbital at around −0.725 eV relative to the Fermi level becomes noticeably more delocalized over both the molecule and the dvGNR device as the bias increases up from 0 to 0.8 V. This increased delocalization indicates stronger coupling between the orbital and the electrodes, which naturally enhances its role in electron transport. Therefore, the enhanced electrostatic interactions between the nucleobase and the nanochannel device at biases between 0.6 and 0.8 V are responsible for the higher electric current values observed in dvGNR and swGNR devices, leading to improved electric current sensitivity, which is less pronounced in prGNR devices.

We also did the sensitivity analysis and the sensitivity is calculated using the formula

8 $$S\%=\left|\frac{I-I_{0}}{I_{0}}\right|\times 100\%$$

where *I* represents the electric current of the GNR+nucleobase devices (nucleobase: A, G, T, and C), and *I*_0_ is the reference electric current of the GNR (i.e, prGNR, dVGNR, and swGNR) devices without any nucleobase. The sensitivity analysis, as shown in Figure 10d–f, provides insight into the performance of each device in detecting different nucleobases. At a bias of 1.8 V (Figure 10d), the prGNR device shows relatively uniform sensitivity across all nucleobases (A, G, T, C), with minor variations. This uniform sensitivity correlates with the observed *I*–*V* response, where all nucleobases exhibited similar electric current trends. The higher applied bias allows the prGNR to detect nucleobases, but the lack of significant differentiation in electric current values suggests that its sensitivity to different nucleobases is limited.

The dvGNR device, at a bias of 0.8 V (Figure 10e), exhibits a highly variable sensitivity, with cytosine (C) showing the highest sensitivity, followed by adenine (A) and thymine (T), while guanine (G) shows the least sensitivity. This variability is consistent with the observed *I*–*V* response, where the dvGNR device displayed distinct electric current profiles for each nucleobase. The pronounced sensitivity, particularly toward cytosine, highlights the dvGNR’s superior ability to distinguish between different nucleobases, likely due to the strong interaction between the localized MOs of the nucleobases and the π-band of the dvGNR, as seen in (Figure 7). The swGNR device, also at a bias of 0.8 V (Figure 10f), shows a more uniform but slightly lower sensitivity compared to the dvGNR device. Cytosine (C) again shows the highest sensitivity, but the differences between nucleobases are less pronounced. This corresponds with the *I*–*V* response, where the swGNR device exhibited distinguishable, but less distinct, electric current profiles for each nucleobase compared to the dvGNR device. The swGNR device is effective in differentiating nucleobases but performs slightly less efficiently than the dvGNR device, likely due to less pronounced *Fano resonances* in the valence band.

It is noted that the prGNR device demonstrates better sensitivity than the dvGNR device only at zero bias, as observed in our current work and reported previously.^32^ However, this performance changes when a bias voltage is applied. In the prGNR device, the electric current increases gradually with bias from 0.5 to 1.8 V (Figure 10a), with all nucleobases exhibiting similar current trends and only minor variations. This limits the ability to distinguish between them under these conditions. In contrast, the dvGNR device (Figure 10b) shows more pronounced differences in current values, particularly at an applied bias of 0.8 V. Beyond 0.5 V, the distinct current profiles for each nucleobase enable effective differentiation.

Further, comparing the three devices, the dvGNR device exhibits the highest current sensitivity and the most distinct *I*–*V* responses across different nucleobases, making it the most effective for nucleobase detection. The prGNR device, while capable of detecting nucleobases at higher biases, lacks the sensitivity and differentiation seen in the dvGNR and swGNR devices. The swGNR device offers balanced performance, with distinguishable *I*–*V* responses and moderate sensitivity, making it a viable option, though still less effective than dvGNR for precise nucleobase differentiation.

The solution environment can play an important role in such studies. Feliciano and co-workers^82^ investigated the effect of a solvent–specifically water–on the zero-bias transmission of a graphene-nucleobase system. Their results showed that water molecules induce a slight change in transmission due to screening effects. This primarily impacts the amount of transverse electrical current, which may increase or decrease depending on the solvent conditions. However, the overall trend or pattern in transmission across different nucleobases remains unchanged. Similarly, studies by Lagerqvist and co-workers^83,84^ looked at how structural fluctuations in nucleotides affect transmission. They found that while these fluctuations do cause changes, the direct influence of water on the transmission can be mostly ignored. In summary, identifying nucleotides based on transverse electrical current is still completely valid, whether or not a solvent is present. The solvent might shift the current values, but it does not alter the distinguishing trends between different nucleotides. In addition, our simulations show conductance changes around 13%, which may be challenging to detect experimentally due to noise and resolution limits. Real-world noise sources such as thermal fluctuations and instrument limitations are not included in the simulations. Typical experimental noise levels range from 1% to 10%, suggesting that larger conductance changes could be detected, while smaller ones might require advanced techniques like signal averaging or improved device design. Additionally, slowing the translocation speed can help reduce noise and enhance resolution.^85,86^ Finally, there are practical challenges in defect engineering, especially in materials like graphene. These include difficulties in controlling where and how defects form, challenges in accurately identifying defects across different scales, issues with producing materials of consistent quality, the complex ways defects influence material properties, and obstacles related to scaling up production and managing costs.^87–89^ By addressing these real-world challenges, the practical application of graphene–such as in DNA sequencing–becomes more achievable.

Graphene nanoribbons (GNRs) have indeed been realized experimentally using several approaches, including bottom-up chemical synthesis,^44^ surface-assisted polymerization,^90^ unzipping of carbon nanotubes,^91^ and lithographic patterning.^92^ Recent advances have also demonstrated device-level applications, such as GNR field-effect transistors with high on/off ratios and stability under ambient conditions.^5^ These works show that experimental platforms for testing our predictions already exist. Our theoretical study can thus provide a basis for future experimental work in several directions. For instance, systematic stability tests of GNR devices under biologically relevant conditions (aqueous solutions, ionic environments, or elevated temperatures) would be essential for biosensing applications. Transport measurements could be designed to directly probe the Fano resonances and current–voltage signatures we predict for nucleobase adsorption. Additionally, defect engineering has recently emerged as a controllable strategy for tuning GNR electronic properties,^14^ which aligns well with our finding that Stone–Wales and divacancy defects play a crucial role in nucleobase sensitivity. Finally, integrating defect-engineered GNRs into scalable device platforms such as hybrid devices could open realistic pathways toward next-generation sequencing technologies.

### 3.7 Conclusions

This study provides a comprehensive analysis of the impact of structural defects on the structural, electronic, and quantum transport properties of graphene nanoribbon (GNR) devices and their potential applications in DNA sequencing. We have shown that defects significantly influence the nature of the bandgap. prGNR and swGNR exhibit a direct bandgap, while dvGNR demonstrates an indirect bandgap. The calculated binding energy values on various GNR surfaces range from −0.06 to −0.10 eV (PBE), −0.55 to −0.80 eV (PBE-D2), and −0.59 to −0.77 eV (vdW-DF2). These binding energy analyses suggest that the semilocal PBE functional does not adequately account for crucial π–π and van der Waals (vdW) interactions. Therefore, vdW-DF2 and PBE-D2 are more reliable for binding energy calculations when DNA nucleobases adsorb onto the prGNR, dvGNR, and swGNR surfaces. Adsorption of DNA nucleobases causes only minor changes in the bandgap (up to 0.02 eV). The defects enhance π–π interactions, leading to the differential stabilization of molecular orbitals compared to prGNR. Charge transfer analysis highlights significant molecular-to-surface contributions, inducing an interfacial dipole that shifts the electronic states, as confirmed by band structure analysis.

Quantum transport analysis demonstrates that while pristine GNRs exhibit distinct conduction channels, the introduction of defects–such as vacancies and Stone–Wales defects–dramatically modifies their electronic and transport properties. Defects result in localized electronic states interacting with delocalized states, producing pronounced *Fano resonances* characterized by sharp dips in the transmission spectra. When DNA nucleobases interact with GNR devices, each nucleobase induces unique resonance peaks in the transmission function, influenced by the type and location of defects. Conductance sensitivity analysis identifies prGNR as the most promising device for nucleobase detection due to its superior sensitivity and its ability to leverage *Fano resonances* for precise electronic detection. Defected devices also show prominent conductance sensitivity values, suggesting their potential utility in DNA detection. *I*–*V* analysis indicates that dvGNR devices exhibit the highest current sensitivity and the most distinct *I*–*V* responses across different nucleobases, making them particularly effective for nucleobase detection. While prGNR devices can detect certain nucleobases, they perform less consistently due to proportional current increases with higher biases, leading to similar trends. In contrast, swGNR devices successfully distinguish all four nucleobases through distinct current signals observed between 0.6 and 0.8 V applied biases.

These findings are crucial for the development of sensitive biosensing technologies, as the defect-induced *Fano resonances* offer a means to detect and differentiate between nucleobases with high precision. The observed transmission changes upon nucleobase adsorption highlight the potential of defected GNRs as effective components in next-generation DNA sequencing and other nanoscale electronic applications. Future research could further explore the optimization of defect structures and their interactions with different biomolecules to enhance the performance of GNR-based sensors and other electronic devices.

## Supplementary Material



## Notes

Atomic structure of the transport devices, distinct absorption configuration, relative energy values, projected density of states plots, *I*–*V* response curves for the dvGNR device with and without nucleobases, bias-dependent transmission spectra for the dvGNR + G system at 0, 0.5, and 0.8 V, together with molecular orbitals (PDF)
