# Supplementary material for: Defect-Engineered Graphene Nanoribbons for Enhanced DNA Sequencing: A Study of Structural Defects and Their Impact on Nucleobase Interaction and Quantum Transport
Source: J Phys Chem B. 2025 Sep 22;129(39):9862–79. doi: 10.1021/acs.jpcb.5c03247 (PMC12498413; doi:10.1021/acs.jpcb.5c03247)
Supplement: Supplementary file 1 [file jp5c03247_si_001.pdf]

Supporting Information for

Defect-Engineered Graphene Nanoribbons for Enhanced DNA Sequencing: A Study of Structural Defects and Their Impact on Nucleobase Interaction and Quantum Transport

Rameshwar L. Kumawat,[*,†] Sanjiv K. Jha,[*,‡] Benjamin O. Tayo,[*,¶] and C. David Sherrill[*,†]

†*School of Chemistry and Biochemistry, Georgia Institute of Technology, Atlanta, Georgia 30332, United States*

‡*Mathematics and Science Division, GateWay Community College, Maricopa Community Colleges, Phoenix, Arizona 85034, United States*

¶*School of Engineering, University of Central Oklahoma, Edmond, Oklahoma 73034, United States*

E-mail: rameshwarlal1122@gmail.com; sanjiv.jha@gatewaycc.edu; btayo@uco.edu; sherrill@gatech.edu

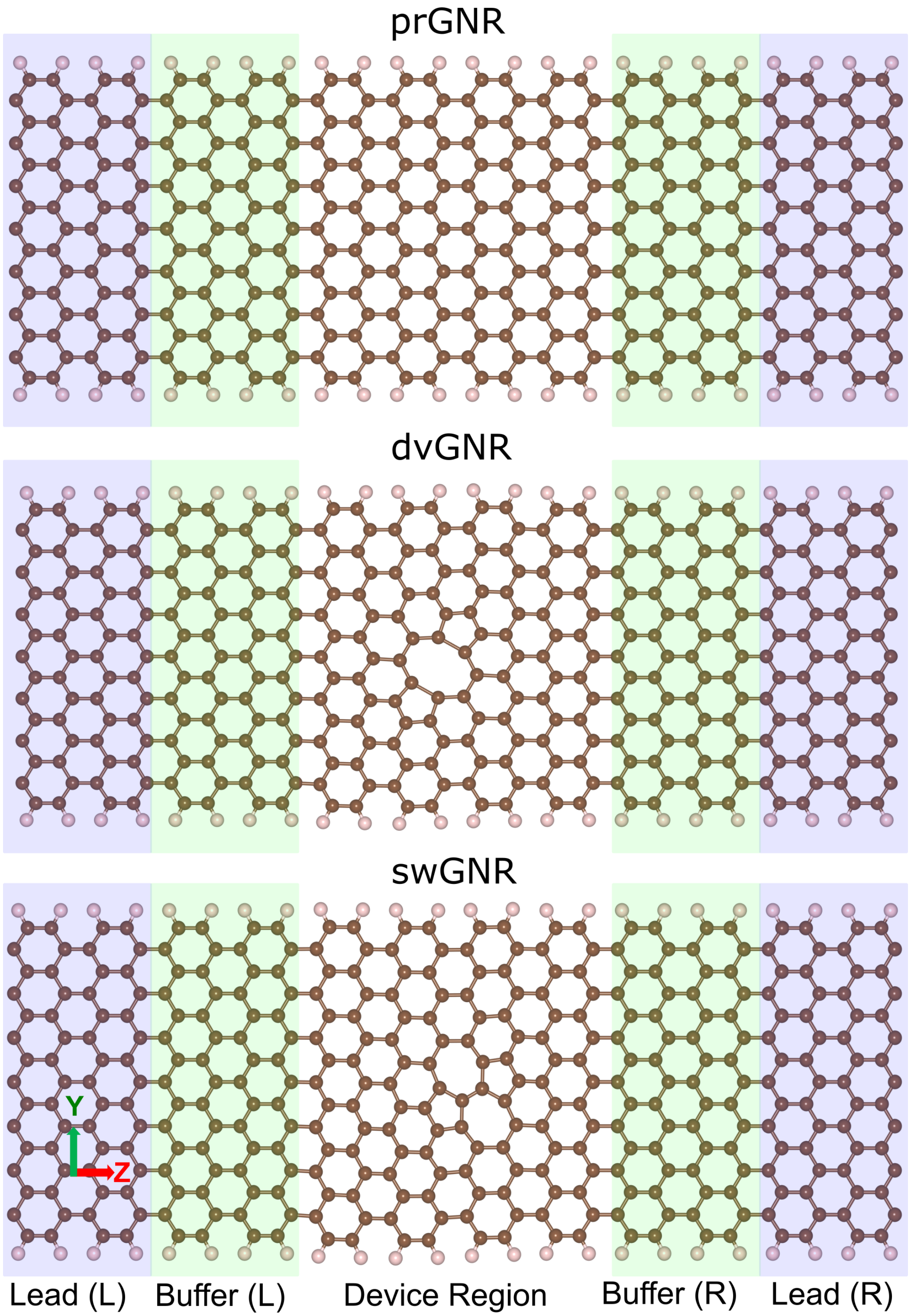

Figure S1: Atomic structures of the transport devices. Illustration of the left (L) and right (R) leads, buffer regions, and the device region. The z-axis represents the transport direction. Color code: C(grey), H (pink).

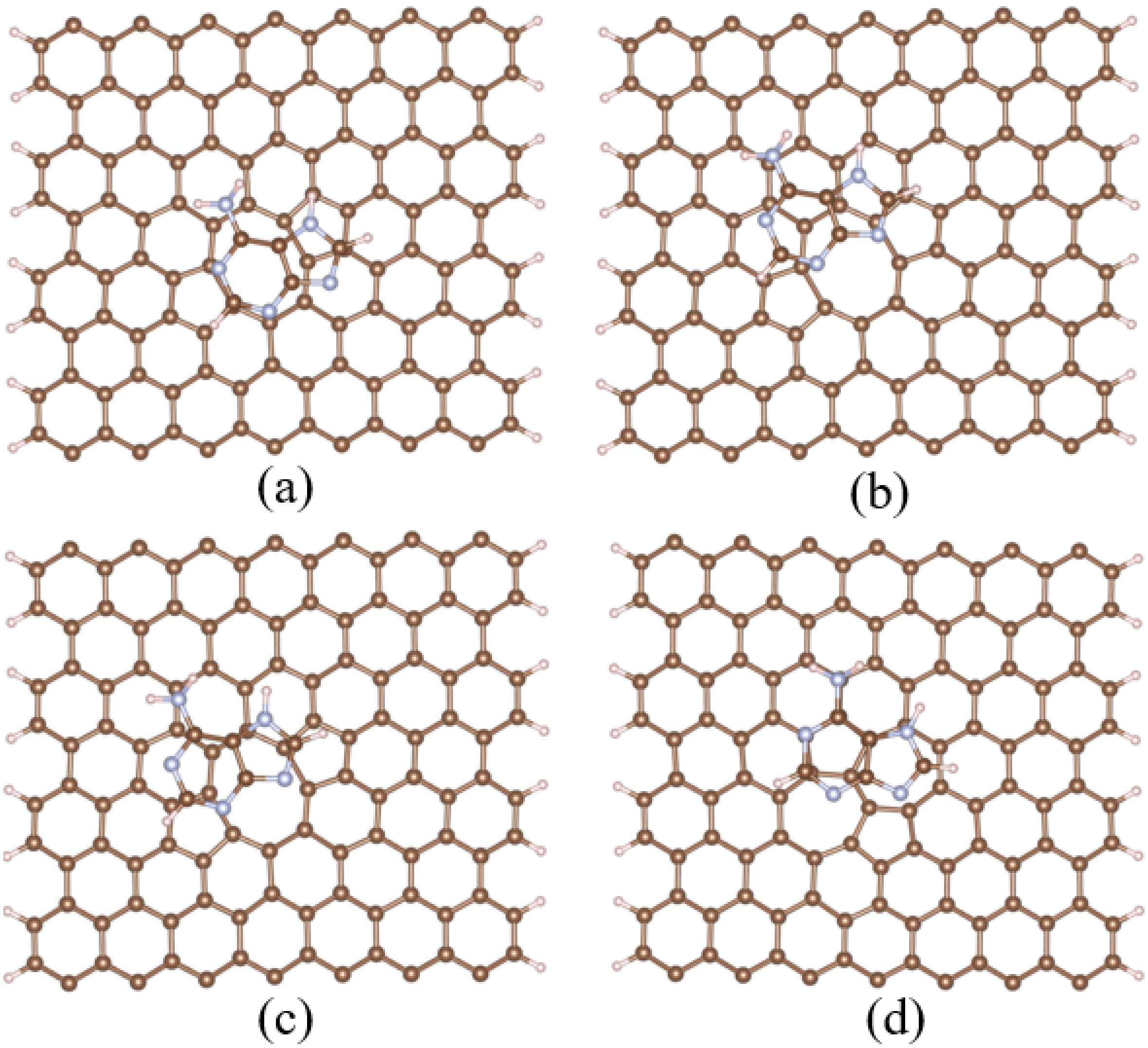

Figure S2: Four distinct absorption configurations illustrated using adenine: (a) C1 (hollow), (b) C2 (top), (c) C3 (bridge), and (d) C4 (center and bridge). Color code: C(grey), H (pink), N (blue).

Table S1: Computed relative energies (in eV) for different GNR systems and their stable configurations.

System	C2	C3	C4	Stable Configuration
prGNR+A	-0.012	0.025		C2
prGNR+G	-0.014	0.028		C2
prGNR+T	0.018	0.033		C1
prGNR+C	-0.014	0.017		C2
dvGNR+A	0.035	0.025		C1
dvGNR+G	0.016	0.014		C1
dvGNR+T	0.018	0.009		C1
dvGNR+C	-0.018	-0.029		C3
swGNR+A	0.009	0.004	0.012	C1
swGNR+G	0.005	0.024	0.020	C1
swGNR+T	0.001	0.026	0.030	C1
swGNR+C	0.002	0.002	0.001	C1

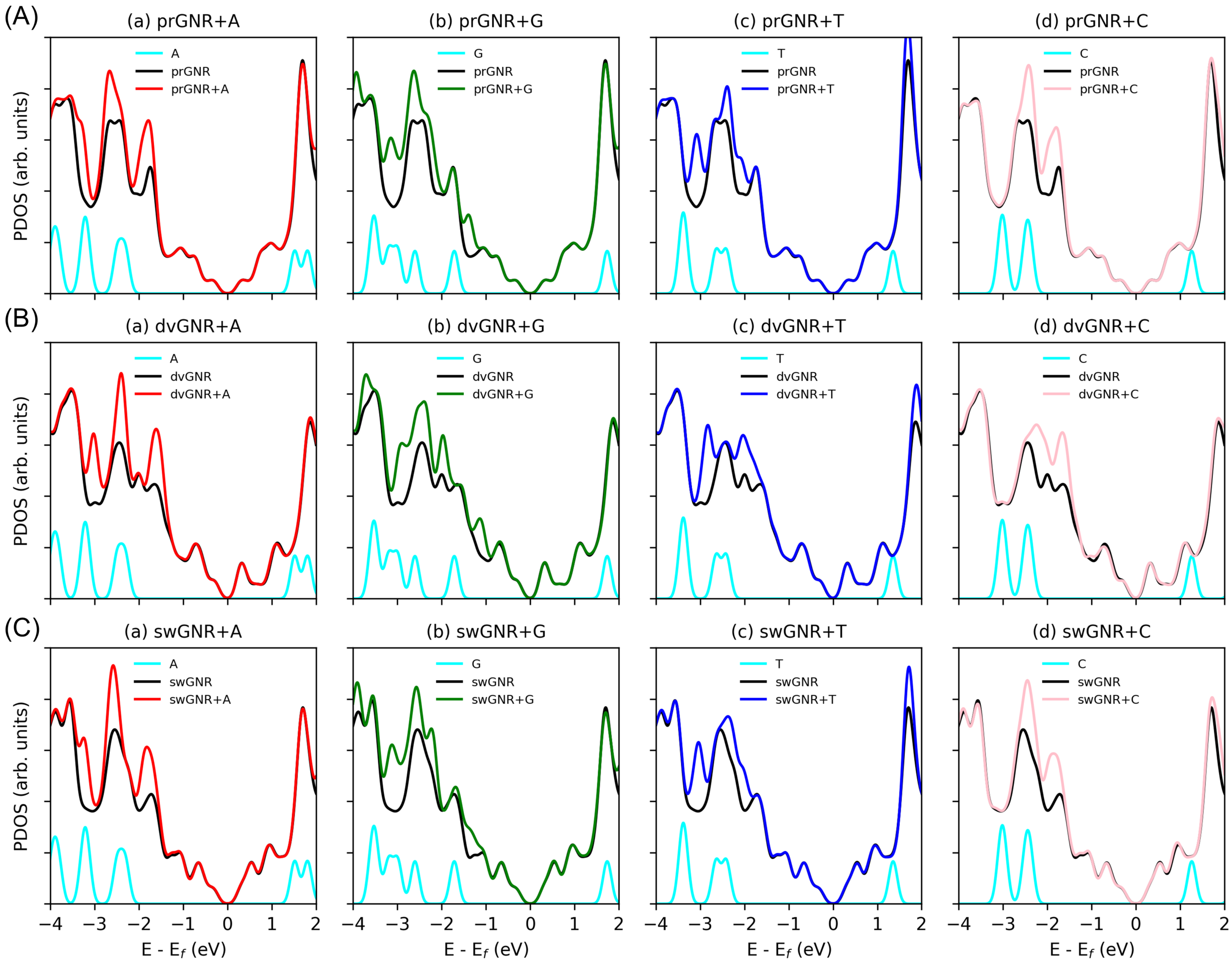

Figure S3: Projected density of states plots for the reference bare substrates (prGNR, dvGNR and swGNR), substrates + nucleobase systems, and bare nucleobases (A, G, T, C) computed using vdW-DF2 functional as described in the Computational details section.

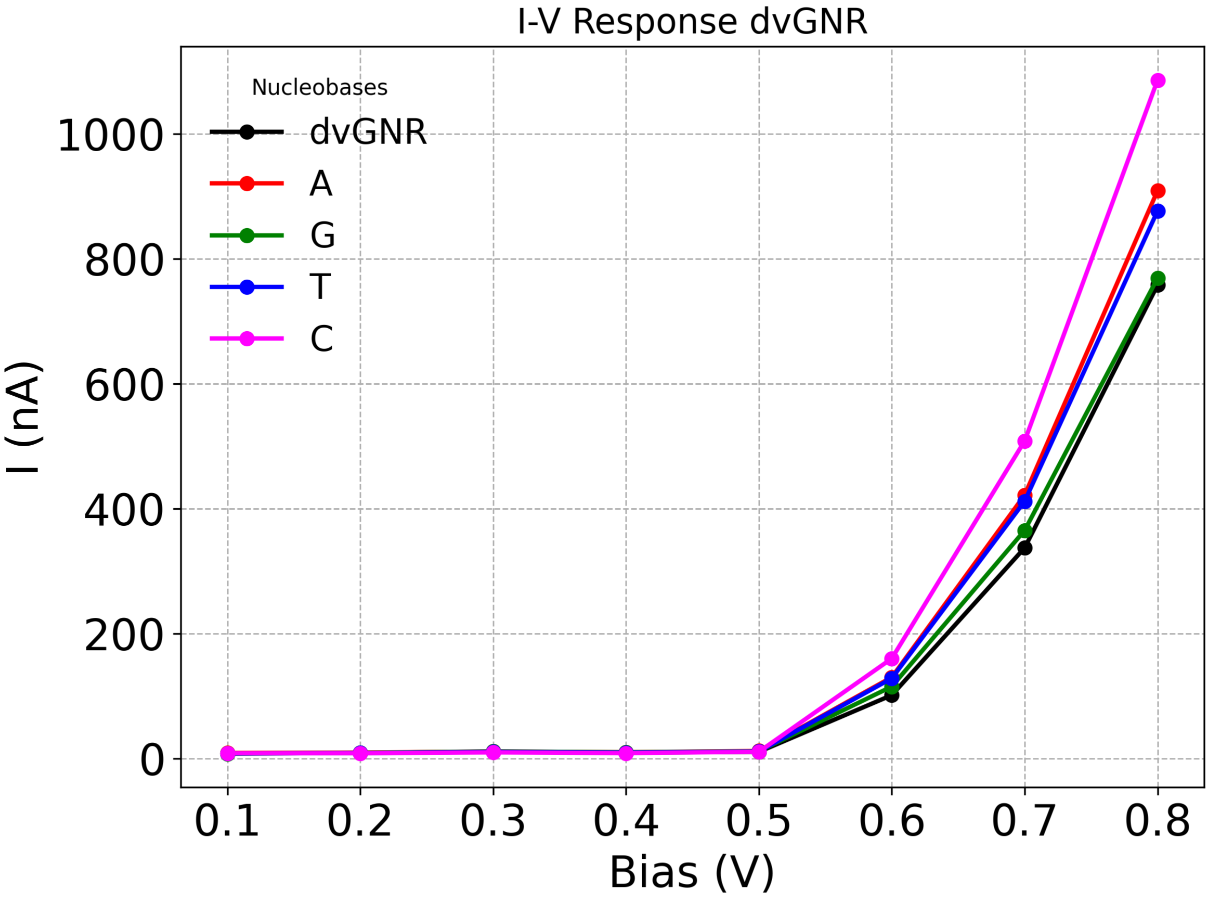

Figure S4: I-V response curves for dvGNR device with and without target nucleobases.

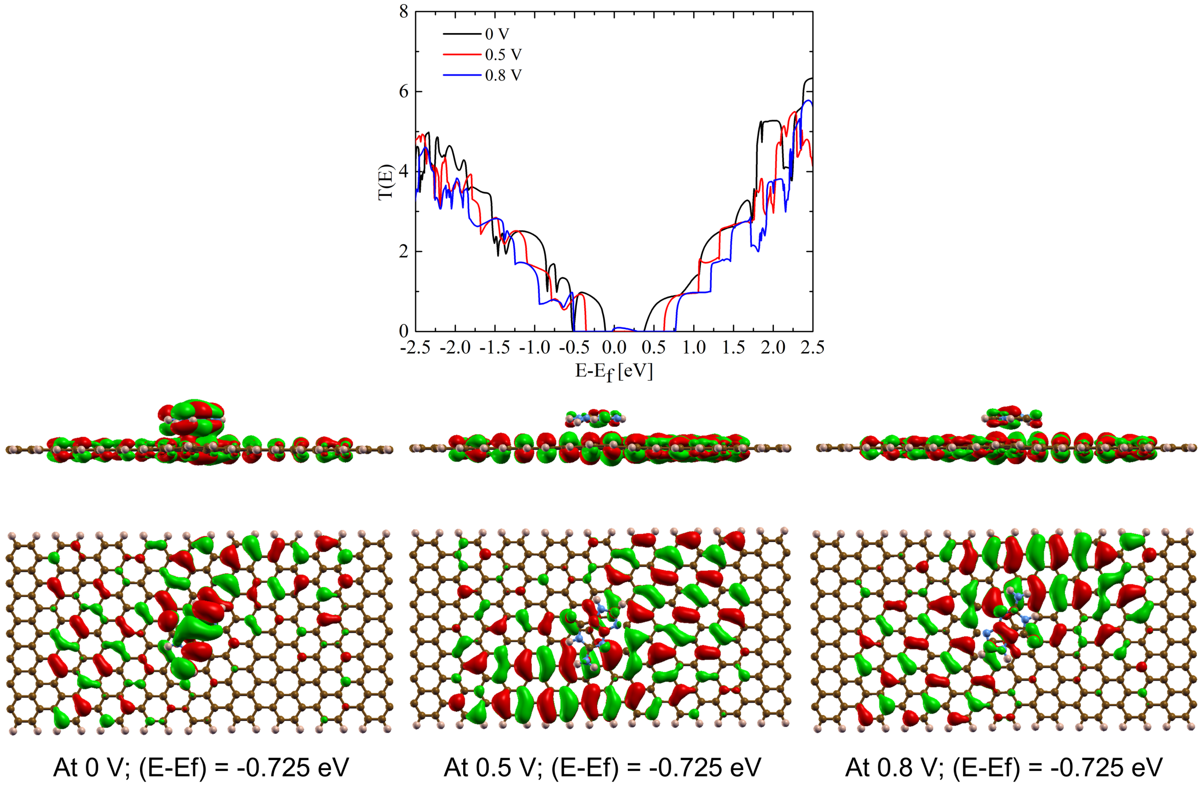

Figure S5: (Top) Bias-dependent transmission spectra under 0, 0.5, and 0.8 V biases, showing transmission relative to the Fermi level. (Bottom) Molecular orbital delocalization at E–E_F = –0.725 eV, showing increased delocalization with bias, suggesting enhanced coupling and charge transport.
